# Enhanced Regional Electric Potential Difference of Graphdiyne Through Asymmetric Substitution Strategy Boosts Li^+^ Migration in Composite Polymer Solid-State Electrolyte

**DOI:** 10.1007/s40820-025-01790-5

**Published:** 2025-05-21

**Authors:** Chao Jiang, Kaihang Wang, Luwei Zhang, Chunfang Zhang, Ning Wang

**Affiliations:** 1 Shandong Provincial Key Laboratory for Science of Material Creation and Energy Conversion, Science Center for Material Creation and Energy Conversion, School of Chemistry and Chemical Engineering, Shandong University, Jinan, 250100 People’s Republic of China; 2 College of Chemistry and Materials Science, Hebei Key Laboratory of Analytical Science and Technology, Hebei University, Baoding, 071002 People’s Republic of China

**Keywords:** Graphdiyne, Asymmetric substitution, Enhanced regional electric potential difference, Solid-state electrolytes, Poly(ethylene oxide)

## Abstract

Low ionic conductivity is a major obstacle for polymer solid-state electrolytes. In response to this issue, a design concept of enhanced regional electric potential difference (EREPD) is proposed to modulate the interaction of nanofillers with other components in the composite polymer solid-state electrolytes (CPSEs). While ensuring the periodic structure of the graphdiyne (GDY) backbone, methoxy-substituted GDY (OGDY) is prepared by an asymmetric substitution strategy, which increases the electric potential differences within each repeating unit of GDY. The staggered distributed electron-rich regions and electron-deficient regions on the two-dimensional plane of OGDY increase the free Li^+^ concentration through Lewis acid–base pair interaction. The adjacent ERRs and EDRs form uniformly distributed EREPDs, creating a continuous potential gradient that synergistically facilitates the efficient migration of Li^+^. Impressively, the OGDY/poly(ethylene oxide) (PEO) exhibits a high ionic conductivity (1.1 × 10^–3^ S cm^−1^) and ion mobility number (0.71). In addition, the accelerated Li^+^ migration promotes the formation of uniform and dense SEI layers and inhibits the growth of lithium dendrites. As a proof of concept, Li||Li symmetric cell and Li||LiFePO_4_ full cell and pouch cell assembled with OGDY/PEO exhibit good performance, highlighting the effectiveness of our EREPD design strategy for improving CPSEs performance.

**Supplementary Information:**

The online version contains supplementary material available at 10.1007/s40820-025-01790-5.

## Highlights

- Methoxy-substituted graphdiyne (OGDY) is synthesized via an asymmetric substitution strategy, featuring a periodic alternation of electron-rich regions and electron-deficient regions, which significantly enhances the heterogeneity of charge distribution in OGDY.
- An enhanced regional electric potential difference design concept is proposed to address the low ionic conductivity of polymer solid-state electrolytes. The OGDY/poly(ethylene oxide) composite polymer solid-state electrolyte achieves an ionic conductivity of 1.1×10^−3^ S cm^−1^ and a high lithium-ion transference number of 0.71.

**Supplementary Information:**

The online version contains supplementary material available at 10.1007/s40820-025-01790-5.

## Graphical abstract

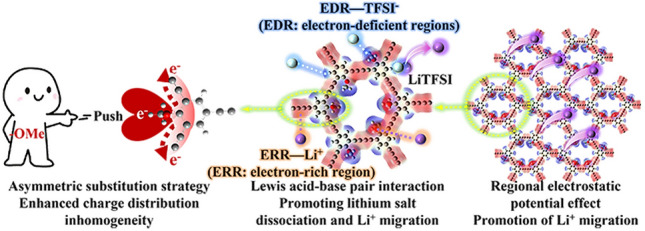

## Introduction

Graphdiyne (GDY) is a revolutionary two-dimensional (2D) carbon allotrope with remarkable structural characteristics [1, 2]. The uniform distribution of *sp* C and *sp*^2^ C in GDY results in an inhomogeneous charge distribution across the 2D carbon plane, generating abundant active sites [3–7]. Consequently, GDY exhibits excellent affinity for a wide range of substances, including metal atoms, ions, nanoclusters, polymers, and organic molecules [8–10]. This fundamental property underpins the outstanding performance of GDY-based materials in various application fields [11–18]. Furthermore, the bottom-up synthesis strategy enables precise and systematic functional group substitution at specific sites of GDY. That can facilitate the further modulation of its local electronic environment and optimization of charge distribution [19–22]. These features endow GDY-based materials with superior performance advantages in complex multicomponent applications [23, 24].

Polymer solid electrolytes (PSEs) are complex multicomponent systems typically composed of the polymer matrix (e.g., poly(ethylene oxide), PEO), lithium salts (e.g., lithium bis(trifluoromethane sulfonyl)imide, LiTFSI), and functional additives [25–29]. To enable practical applications of polymer solid electrolytes, several critical challenges must be addressed: (1) low ionic conductivity (σ), particularly at low temperatures [30, 31]; (2) high interfacial impedance [32, 33]; (3) poor electrochemical stability [34, 35]; and (4) lithium dendrite growth [36, 37]. Among these limitations, the low ionic conductivity remains the most significant drawback of polymer solid electrolytes when compared to their inorganic counterparts [38]. The incorporation of 2D layered materials into PSEs to form composite polymer solid-state electrolytes (CPSEs) has been demonstrated as an effective strategy to enhance the σ, even in the case of low additions [39–42]. Generally, 2D nanofillers with high aspect ratios can effectively reduce the crystallinity of polymers and improve the motility of polymer chain segments, thus improving the migration of Li^+^ in the polymer chain segment [43–45]. With the deepening research of CPSEs, it has been discovered that precisely regulating the interactions among the polymer, lithium salt, and nanofillers can further improve the σ of CPSEs [46, 47]. Ideal 2D nanofillers typically need to possess the following characteristics: (1) strong affinity with anions (such as TFSI^−^) to promote the effective dissociation of lithium salts; (2) moderate interaction with Li^+^ to provide effective Li^+^ binding sites and facilitate Li^+^ transport [48]. Notably, the above interactions are fundamentally related to the electronic structure of 2D nanofillers, particularly the surface charge distribution on the 2D plane [49–52]. Therefore, precise regulation of the charge distribution of nanofillers is crucial for optimizing these interactions and enhancing the overall performance of CPSEs.

Common 2D nanofillers such as MXenes, graphene, and boron nitride (BN) exhibit significant limitations. Their structural modifications typically rely on post-synthesis treatments including acid washing, grafting, and calcination, which often fail to achieve uniform and controllable structural regulation [53–56]. The resulting structural and electronic heterogeneity leads to non-uniform ion transport, hindering lithium-ion migration while accelerating lithium dendrite growth [57]. In contrast, GDY exhibits multiple advantages due to its unique structural characteristics and straightforward synthesis: (1) Its non-uniform charge distribution creates specific binding sites for both Li^+^ and TFSI^−^; (2) The charge distribution can be precisely optimized through targeted functional group modification [20, 58]. These distinctive properties enable GDY to simultaneously enhance the σ and interfacial stability of CPSEs, while serving as an ideal platform for investigating the structure–property relationships and underlying mechanisms in CPSE systems.

Herein, a novel approach was presented to resolving the long-standing challenge of low σ in PSEs by leveraging the concept of enhanced regional electric potential difference (EREPD) to optimize ion transport dynamics. An asymmetric substitution strategy is employed to enhance the local charge distribution heterogeneity in the carbon skeleton of GDY while maintaining its structural order and crystallinity, thereby forming an EREPD. In the designed precursor, the three meta-positions of the benzene ring are substituted with a methoxy group (-OMe) and two hydrogen atoms (-H), leading to the synthesis of methoxy-substituted GDY (OGDY). The OGDY is used to prepare CPSEs OGDY/PEO. In OGDY, oxygen atoms, and diacetylene bonds form electron-rich regions (ERRs), while the substituted hydrogens on the methyl groups and benzene rings form electron-deficient regions (EDRs). The ERRs act as Lewis acids to interact with TFSI^−^, promoting lithium salt dissociation and increasing the number of free Li^+^ ions. The EDRs serve as Lewis bases to bind Li^+^, facilitating Li^+^ migration. The EREPD formed between adjacent EDRs and ERRs is uniformly distributed on the 2D OGDY plane, which provides a fast channel for directional Li^+^ migration. Additionally, the 2D lamellar structure of OGDY and its electrostatic interactions with PEO effectively reduces the crystallinity of OGDY/PEO. This crystallinity reduction significantly enhances the mobility of polymer segments and further promotes Li^+^ migration. The results show that OGDY/PEO achieves a σ of 1.1 × 10^–3^ S cm^−1^ and a Li^+^ transference number (t_Li+_) of 0.71. The accelerated Li^+^ migration also promotes the formation of a dense and uniform SEI, suppressing lithium dendrite growth and improving battery stability. As a proof of concept, the Li|OGDY/PEO|Li symmetrical cell operates stably for 850 h at 0.1 mAh cm^−2^. The Li|OGDY/PEO|LFP full cell is cyclically stabilized for 205 cycles at 0.5C, and the initial specific capacity reaches 158.7 mAh g^−1^ with a capacity retention rate of 91.4%. The pouch cell can run stably even under the conditions of bending, and folding, showing a good practical application prospect.

## Experimental Section

### Materials and Characterization Methods

The reagents used in the experiments are analytically pure reagents purchased from reagent suppliers. Pyridine (≥ 99.5%) and tetrahydrofuran (THF, ≥ 99.5%) were purchased from Sinopharm Chemical Reagent Co., Ltd. (Shanghai, China). Ethyl acetate (≥ 99.5%), N, N-dimethylformamide (DMF, ≥ 99.5%), and anhydrous ethanol (≥ 99.7%) were obtained from Tianjin FuYu Fine Chemical Co., Ltd. (Tianjin, China). Triethylamine (≥ 99.5%), tetrabutylammonium fluoride (TBAF, 1 M in THF), trimethylsilylacetylene (98%), super dry acetonitrile (99.9%), and lithium bis(trifluoromethanesulfonyl)imide (LiTFSI, 98%) were supplied by Energy Chemical (Shanghai, China). Hydrochloric acid (HCl, 36–38%), acetone (≥ 99.5%), and toluene (≥ 99.5%) were acquired from Yantai Far Eastern Fine Chemical Co., Ltd. (Shandong, China). Poly(ethylene oxide) (average Mv ~ 600,000, powder) was procured from Aladdin Biochemical Technology Co., Ltd. (Shanghai, China). 2,4,6-Tribromoanisole (98%) was purchased from Macklin. Organic solvents such as THF and toluene are redistilled, and LiTFSI and PEO are dried. The materials used for assembling the cells such as lithium flakes, aluminum foil, and battery cases are also purchased commercially. The lithium foil (100 μm, ≥ 99.9%) was purchased from Kejing Star Technology Co., Ltd. (Shenzhen, China). The aluminum foil (20 μm, ≥ 99.6%) and battery casings were obtained from Canrd New Energy Technology Co., Ltd. (Guangdong, China).

Conformal characterization was carried out by Gemini SEM 300 scanning electron microscope (SEM). The solid electrolyte films were tested by X-ray powder diffractometer (XRD) operated at Cu Kα, 2.2 KW. The test angles were 5°-90°. Elemental valence analysis was tested by X-ray photoelectron spectroscopy (XPS) with a Thermo Fisher Scientific-ESCALAB 250Xi, USA. The thermal stability of the solid electrolyte films was tested by a thermogravimetric analyzer (Netzsch-TG 209F3, Germany) at room temperature up to 600 °C with a ramp rate of 10 °C min^−1^. The glass transition temperature of the solid electrolyte films was determined by a simultaneous thermal analyzer. The temperature range of the test was from room temperature to 800 °C with a ramp rate of 10 °C min^−1^. Raman spectra were measured by a HORIBA PHS-3C Raman spectrometer with an excitation wavelength of 473 nm. The Raman spectra of PEO and OGDY/PEO were deconvoluted based on the characteristic peaks of free TFSI^−^ (736–741 cm^−1^) and bonded TFSI^−1^ (743–750 cm^−1^) [59]. The percentage of free TFSI^−^ was calculated using the following equation:

1 $$\left( {{\text{Free}}\;{\text{TFSI}}^{ - } } \right){\text{\% }} = \frac{{{\text{A}}_{{{\text{free}}\;{\text{TFSI}}^{ - } }} }}{{{\text{A}}_{{{\text{free}}\;{\text{TFSI}}^{ - } }} + {\text{A}}_{{{\text{Ion}}\;{\text{cluster}}}} }} \times 100\%$$

where A_free TFSI-_ is the deconvoluted peak area of free TFSI^−^ and A_Ion cluster_ is the deconvoluted peak area of bound TFSI^−^. ^7^Li solid-state magic-angle-spinning (MAS) nuclear magnetic resonance (NMR) spectra were recorded on an Ascend 600 MHz WB AVANCE NEO spectrometer. Stress–strain tests were performed on an Instron universal material testing machine.

### Synthesis

#### Synthesis of ((2-methoxybenzene-1,3,5-triyl)tris(ethyne-2,1-diyl))tris(trimethylsilane) (Compound 1)

Dissolve 3 g of tribromoanisole in 100 mL of toluene, then pour into 100 mL of triethylamine and stir well. To the reaction solution, 20 mL of trimethyl ethynyl silyl, 0.364 g of CuI, and 0.541 g of Ph_3_P were added. The mole ratio of tribromoanisole/trimethyl ethynyl silyl/CuI/Ph_3_P is 8:1:7:1. The reaction was carried out at 80 °C for 5 days under an Ar atmosphere. The product obtained was sequentially separated and purified by filtration and column chromatography to obtain a light yellow oil. The structure of compound (1) is shown in Fig. S1. And the structure was determined by ^1^H and ^13^C NMR spectroscopy (Figs. S2 and S3).

#### Synthesis of OGDY

Hundred milligrams of compound (1) was dissolved in 50 mL of tetrahydrofuran (THF) to prepare a solution with a concentration of 5 mM. Then, 1 mL of tetrabutylammonium fluoride (TBAF, 1 M in THF) was added. The molar ratio of TBAF/compound (1) is 1:4. The reaction was carried out in an Ar atmosphere at 0 °C without light for 30 min. The solution was poured into a partition funnel and washed by 3 extractions with ethyl acetate and saturated saline. Then, anhydrous Na_2_SO_4_ was used to remove water from the upper organic phase. This organic phase solution was spin-dried to give a white solid (1,3,5-triethynyl-2-methoxybenzene (2)).

The solid was dissolved in 100 mL of pyridine (2.5 mM) and poured into two 100-mL autoclaves. Four pieces of copper foil (2 cm × 10 cm) with an oxide layer removed were placed into the two Teflon-lined autoclaves. The Teflon-lined autoclaves were assembled and placed in an oven at 110 °C for a hydrothermal reaction for 12 h. A yellow film grew on the surface of the copper foil after the reaction. The copper foil was soaked in ammonia and ultrasonically stripped of the OGDY film. The obtained OGDY was ultrasonically washed with ammonia to remove copper ions. Then, it was boiled and washed with N, N-dimethylformamide (DMF) to remove oligomers. Finally, the filtered solids were rinsed with ethanol and dried overnight in an oven at 60 °C.

#### Synthesis of triacetylene monomer (tris(trimethylsilyl)ethynylbenzene) (Compound 2)

Pour 220 mL of redistilled THF into a three-necked flask, pass Ar, stir for 15 min, and ice-acetone bath temperature control -78 °C. Add 42 mL of TMSA. 140 mL of n-butyllithium was added by a constant pressure dropping funnel with a dropping rate of 2 drops s^−1^, and the reaction was carried out at a low temperature for 1 h. Fifty-six grams of anhydrous ZnCl was poured in, and the reaction was carried out at a low temperature for 30 min, followed by 3 h of reaction at room temperature. 8.8 g of tribromobenzene, 350 mg of Pd(PPh_3_)_4_, and 220 mL of toluene were poured in, and the reaction was carried out at 80 °C for 3 days. Pour in 3 M HCl 200 mL, and stir to dissolve the solid. Extraction was washed with ethyl acetate with saturated saline, and the organic phase was poured into anhydrous NaSO_4_ for drying, distilled under reduced pressure to obtain a yellow solid crude product, recrystallized and purified by column chromatography to obtain a white solid product.

#### Symmetrically Hydrogen-substituted GDY (HGDY) Polymerization

A three-necked flask was poured with 50 mL of THF; 150 mg of monomer (compound (2)) was added, dissolved with stirring, passed through Ar, ice-acetone bath, and temperature-controlled at 0 °C. The reaction was carried out with 15 mL of TBAF for 30 min. Inject 15 mL TBAF and react for 30 min without light. Extract and wash with ethyl acetate and saturated saline, the organic phase was poured into anhydrous NaSO_4_ for drying. Decompression distillation was performed to obtain triethynylbenzene. Two hundred and forty milliliters of pyridine was dissolved solid and poured into three 100-mL high-pressure reactors, respectively. Twelve pieces of 2 cm × 10 cm copper foils were put into 3 M HCl solution and ultrasonicated for 15 min, rinsed with distilled water, anhydrous ethanol, and acetone at a time, and finally blown dry by Ar gas. Four pieces of treated copper foil were put into each of the three reactors. The assembled autoclave was placed in an oven at 110 °C for 12 h. The copper foils were removed, rinsed twice with anhydrous ethanol, and soaked in 3 M HCl.

#### Preparation of PEO PSE

Twelve milligrams of anhydrous acetonitrile, 0.244 g of LiTFSI, and 0.6 g of PEO were added to a 20-mL glass vial and stirred at 800 r min^−1^ for 24 h. Four milliliters of the viscous solution obtained was poured into a flat-bottomed Teflon evaporation dish with a diameter of 6 cm and left horizontally in a glovebox for 4 h. Finally, the pre-dried solid electrolyte film was put into a vacuum oven at 60 ℃ for 18 h. The dried membrane is perforated into a solid electrolyte membrane with a diameter of 2 cm for battery assembly.

#### Preparation of OGDY/PEO and HGDY/PEO CPSEs

Twelve milligrams of OGDY powder and 12 mL of anhydrous acetonitrile were added to a 20-mL glass vial, ultrasonically dispersed for 2 h, and stirred for 12 h. 0.244 g of LiTFSI and 0.6 g of PEO were added to the OGDY/acetonitrile suspension and stirred for 24 h. 4 mL of the viscous solution obtained was poured into a flat-bottomed evaporation dish of Teflon with a diameter of 6 cm, and left horizontally in the glove box for 4 h. Finally, the pre-dried solid electrolyte film was dried in a vacuum drying oven at 60 °C for 18 h. The dried film was perforated to form a solid electrolyte film with a diameter of 2 cm for battery assembly. The preparation of HGDY/PEO CPSEs refers to the preparation method of OGDY/PEO.

#### Preparation of LiFePO_4_ (LFP) Cathode

LFP, conductive carbon black, and polyvinylidene difluoride (PVDF) were added to an agate mortar in the mass ratio of 8:1:1. N-methylpyrrolidone (NMP) was added drop by drop and ground for 30 min to make a homogeneous mixture. The LFP slurry was uniformly coated on the Al foil using a 100 μm tetrahedron. The slurry-coated Al foil was dried in a vacuum drying oven at 120 °C for 24 h and transferred to a glove box filled with argon gas. The dried Al foil was perforated to form a 1-cm-diameter anode sheet. LFP loading is about 3 mg cm^−2^.

### Battery Assembly and Testing

Li||Li and Li||LFP full cells with different solid electrolyte films were assembled in an argon-filled glove box. The lithium negative electrode was a lithium foil with a diameter of 1 cm. The cell encapsulation pressure was 500 psi. The assembled cells were tested for long-cycle stability and multiplicity performance by a multichannel battery test system. The battery was tested at a temperature of 60 °C. The Li||LFP was tested over a voltage range of 2.8 to 4.0 V.

### Electrochemical Performance Measurements

Σ was tested by electrochemical impedance spectroscopy (EIS). The fabricated solid electrolyte membrane was sandwiched between two parallel stainless sheets to assemble a barrier cell. The resistance (R) was measured at 30, 40, 50, 60, 70, 80, 100, and 150 °C using an electrochemical workstation at an open-circuit voltage, frequency of 1000 kHz–0.1 Hz, and amplitude of 10 mV. The σ was calculated by Eq. 2, where R_b_, L, and S are the resistance (Ω), thickness (cm), and area (cm^2^) of the solid electrolyte membrane, respectively.

2 $$\sigma =\frac{L}{{R}_{b}\cdot S}$$

The electrochemical stabilization window of the prepared solid electrolyte membrane was tested using linear scanning voltammetry (LSV) technique. The solid electrolyte film was sandwiched between stainless steel and lithium sheets to form the cell. The LSV tests were performed on a CHI660E electrochemical workstation at 60 °C with a scan rate of 0.2 mV s^−1^ and a scan range of 3–6 V. The cells were then subjected to a LSV technique.

Alternating current impedance and direct current polarization tests were performed on Li||Li pair batteries to calculate their t_Li+_. The calculation formula is as follows:

3 $${t}_{L{i}^{+}}=\frac{{I}_{s}\left(V-{I}_{i}{R}_{i}\right)}{{I}_{i}\left(V-{I}_{s}{R}_{s}\right)}$$

where I_s_ and I_i_ are the steady-state and initial current values, respectively, V is the applied voltage, and Ri and Rs are the electrolyte/electrode interfacial resistance measured before and after DC polarization, respectively.

The σ of PEO and OGDY/PEO at different temperatures conform to the classical Arrhenius formula:

4 $$\sigma T = A\exp \left( {\frac{{ - E_{a} }}{RT}} \right)$$

where T is the thermodynamic temperature; A is the preexponential factor; E_a_ stands for the activation energy (kJ mol^−1^); R is the gas constant (8.314 J mol^−1^ K^−1^). The activation energies of PEO and OGDY/PEO were calculated according to this formula.

### Computational Details

Calculations based on first-principles density functional theory (DFT) for the binding energy of Li^+^ absorbed on different sites of OGDY and the NPA charges were carried out by B3LYP functional [60] and 6-311G** basis set [61] as implemented in the Gaussian 09 package [3], where all the structures were fully optimized firstly and followed by subsequent frequency calculations. Zero number of imaginary frequencies was obtained confirming the true minima of these structures. The binding energies were defined as the energy difference between the total Gibbs free energy of bonded species AB and the sum of energies of individuals A and B, the BE can be calculated by Eq. 5:

5 $${\text{BE}} = {\text{EG}}\left( {{\text{AB}}} \right) - {\text{EG}}\left( {\text{A}} \right) - {\text{GE}}\left( {\text{B}} \right)$$

More calculations based on DFT calculations and molecular dynamics simulations were executed utilizing the Vienna Ab initio Simulation Package (VASP) [62] in conjunction with the Projector Augmented Wave (PAW) methodology [63]. The exchange–correlation functional was managed within the parameters of the Generalized Gradient Approximation (GGA), adopting the Perdew–Burke–Ernzerhof (PBE) functional [64]. The long-range van der Waals interactions are accounted for through the DFT-D3 approach [65]. The sampling of the Brillouin zone was conducted using a 1 × 1 × 1 Gamma point grid in order to keep the computational cost affordable. We implemented a plane wave basis set with an energy cutoff set at 500 eV, and the geometric relaxation was carried through until the forces acting on each atom were less than 0.05 eV Å^−1^. To assure rigorous consistency, calculations were performed until the energy convergence threshold was less than 10^–5^ eV.

Both the ab initio molecular dynamics (AIMD) and Slow-growth approach simulations were sampled within the canonical (NVT) ensemble by Nosé–Hoover thermostats with a time step of 1.0 fs at a finite temperature of 300 K. We mainly adopted the Slow-growth method [66, 67] to obtain the migration barrier of Li ion. In the Slow-growth method, a geometric parameter *λ*, i.e., the collective variable (CV), is constrained during the dynamics and varied from the value characteristic of the initial state (*λ*_0_) to that of the final state (*λ*_1_) with a velocity of transformation ʋ. The Helmholtz free energy difference Δ*F* can be computed as Eq. (6) by collecting the derivative of the potential energy with respect to lambda during the simulation, and by integrating over lambda afterward.

6 $$\Delta F = \mathop \smallint \limits_{{\lambda_{0} }}^{{\lambda_{1} }} \left( {\frac{\partial V\left( q \right)}{{\partial \lambda }}} \right) \cdot \upsilon {\text{d}}t$$

In the limit of infinitesimally small *ʋ*, ΔF corresponds to the free energy difference Δ*G* between the final and initial state. The parameters of the method for different migration paths are presented in Tables S1.

## Results and Discussion

### Synthesis and Characterization of the 2D Nanofiller OGDY

**Fig. 1:**
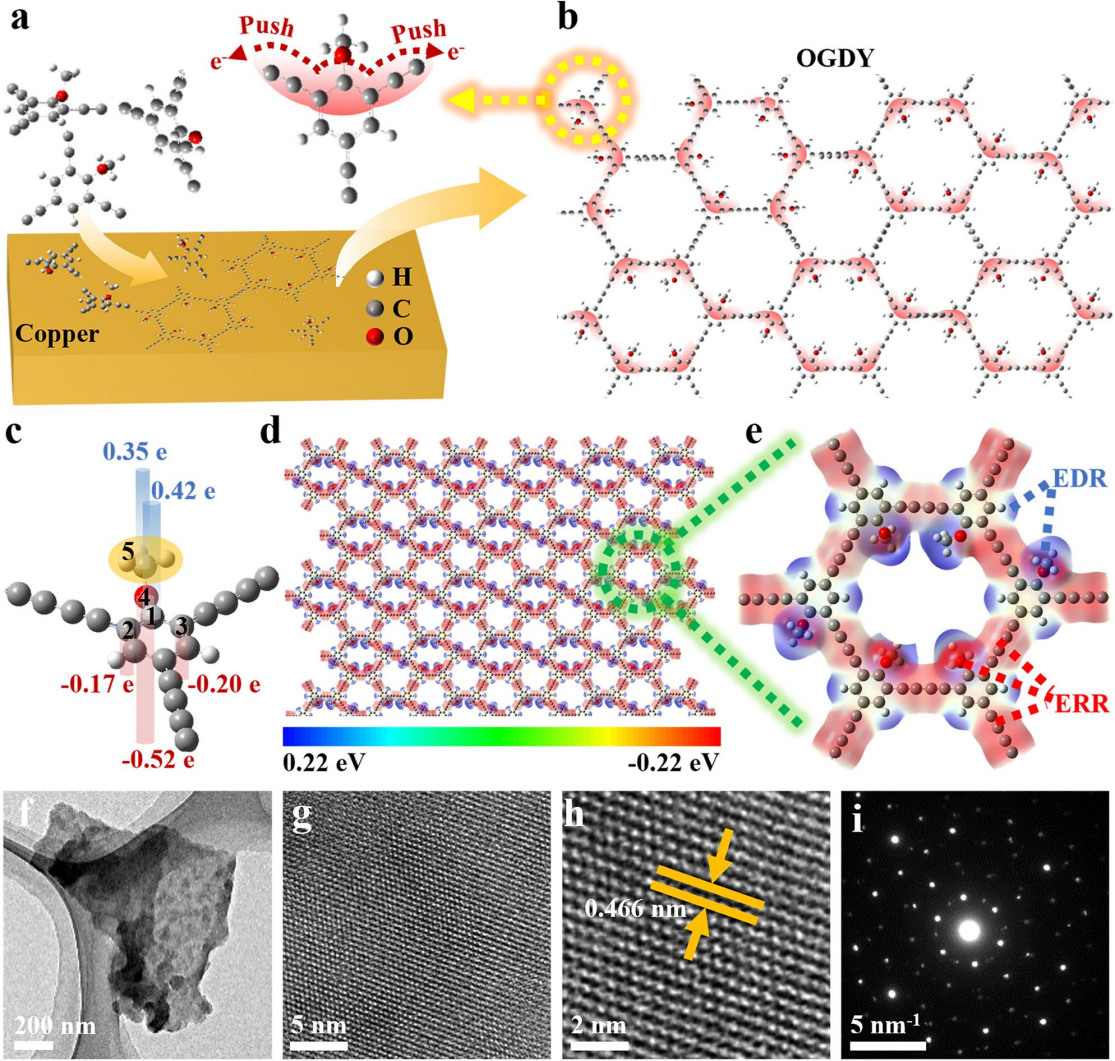
**a** Schematic of the preparation of OGDY. **b** Schematic representation of the electron-donating effect of methoxy on the distribution of the OGDY charge. **c** NPA charge of OGDY. **d** EPS of 2D lamellar OGDY. **e** Enlarged view of the localized EPS of OGDY. EDRs and ERRs refer to blue, and red areas, respectively. **f–h** HRTEM images, **i** the selected area electron diffraction (SAED) of OGDY

The schematic synthesis of OGDY is depicted in Fig. 1a. The GDY precursor asymmetrically substituted by methoxy groups was designed and synthesized, where three meta-positions of the benzene ring were substituted with one -OMe and two -H atoms, respectively (Figs. S1–S3). Then the acetylene bonds were connected through a Glaser-Hay cross-couple reaction to generate the 2D OGDY (Fig. 1b). Our design idea is to use the electron-donating effect of the -OMe and the conjugation effect of the benzene ring to form a significantly enhanced charge distribution asymmetry in the OGDY framework.

To investigate the effect of asymmetrically substituted methoxy on the charge distribution of OGDY, the natural population analysis (NPA) of atomic charges in OGDY was performed (Fig. 1c and Table S2). The electron-donating effect of the -OMe group shifts electrons from the carbon atom on the benzene ring attached to the methoxy group (site 1) to the two adjacent carbons (sites 2 and 3). The charge on site 1 is + 0.42 e, while the charges on sites 2 and 3 are − 0.17 and − 0.20 e, respectively. Additionally, the charge on the oxygen atom (site 4) is − 0.52 e, while the charge on the methyl group (site 5) is + 0.35 e. This analysis reveals a highly heterogeneous charge distribution within the repeating unit, with a localized charge difference of up to 0.94 e. The electrostatic potential surface (EPS) (Fig. 1d, e) shows the differentiated distribution of charges within the OGDY 2D network more visually. In the EPS diagram, substituted hydrogen atoms on the benzene ring and the methyl group are positively charged and represent electron-deficient regions (EDRs). By contrast, the oxygen atoms and the diacetylene bond are negatively charged, forming electron-rich regions (ERRs). There is an EREPD (~ 0.44 eV) between neighboring EDR and ERR. The regular arrangement of OGDY repeating units in the 2D plane allows the EDRs and ERRs to alternate in the OGDY 2D network plane. Furthermore, the EREPDs between adjacent ERRs and EDRs are uniformly distributed on the 2D OGDY plane.

In addition, the morphology and structure of the synthesized OGDY were fully characterized. The XRD pattern of OGDY shows one broad characteristic diffraction peak at 20.9° (Fig. S5). As shown in the HRTEM images, OGDY has clear lattice fringes with a lattice spacing of 0.466 nm (Fig. 1f–h). In addition, its diffraction pattern is a regular hexagon, which attests to crystalline ordering in localized regions (Fig. 1i). Moreover, the structure of OGDY was also characterized by SEM, FTIR, and Raman (Fig. S4). It can be seen that the periodic structure and crystallinity of the synthesized OGDY are well maintained.

### Morphology, Thermal, and Mechanical Properties of OGDY/PEO and PEO

**Fig. 2:**
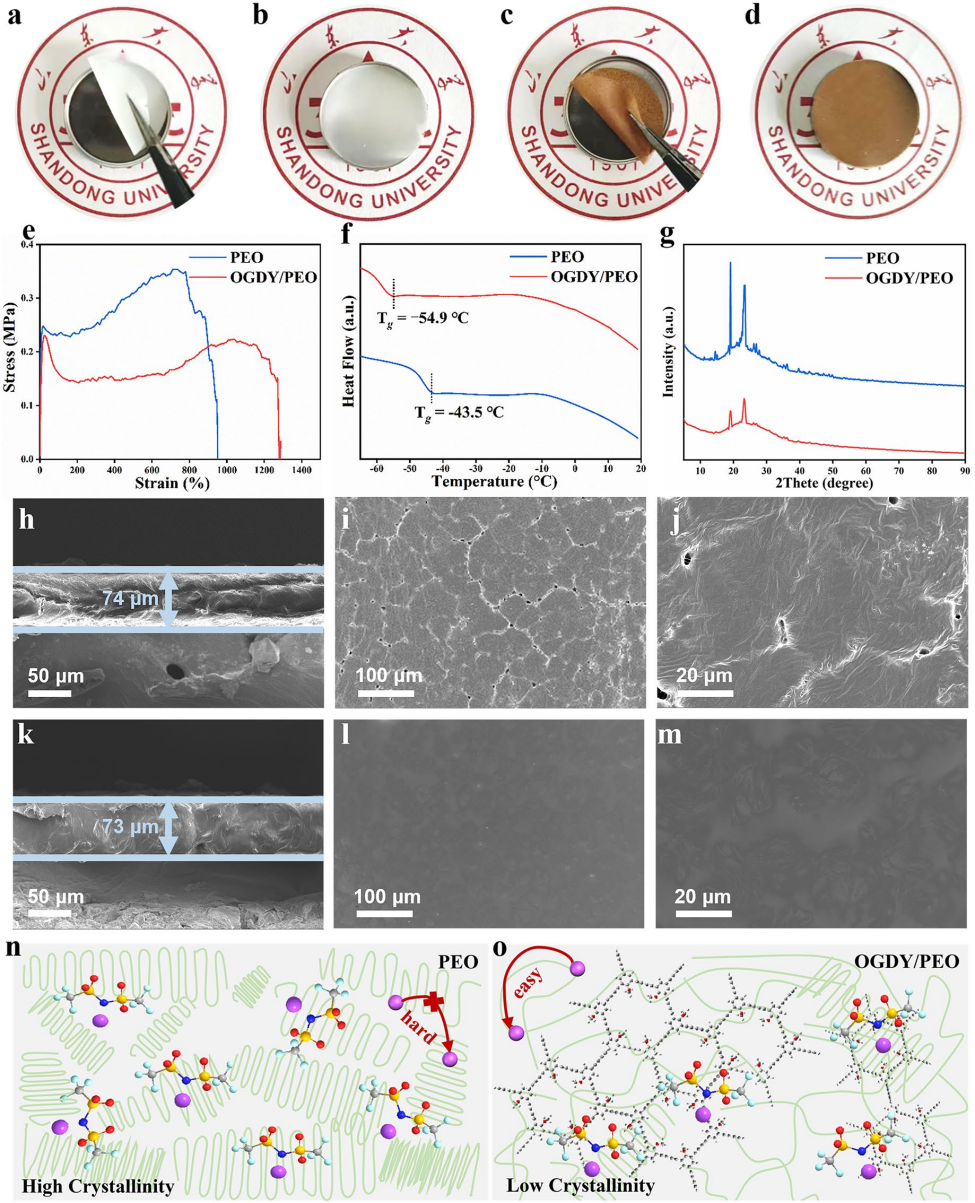
Morphology, thermal, and conductivity properties of PSEs. Optical photograph of **a, b** PEO and **c, d** OGDY/PEO at room temperature (25 °C). **e** Stress–strain curves of PEO and OGDY/PEO. **f** DSC of PEO and OGDY/PEO membranes. **g** XRD patterns of PEO and OGDY/PEO. Cross-section SEM images of **h** PEO and **k** OGDY/PEO. Top-view SEM images of **i, j** PEO and **l, m** OGDY/PEO at various magnifications. Schematic representation of Li^+^ migration in **n** high crystallinity PEO and **o** low crystallinity OGDY/PEO

The pure PEO PSE and OGDY/PEO CPSE were fabricated using the solvent casting method (Fig. S6), followed by comprehensive characterization and analysis of their morphology, thermal properties, and mechanical performance. The white translucent PEO PSEs (Fig. 2a, b) and the yellow–brown OGDY/PEO CPSEs (Fig. 2c, d) both can be bent freely at room temperature, indicating that CPSE can still maintain good flexibility after the introduction of OGDY. TGA and heating experiments show that OGDY/PEO has better thermal stability than PEO (Fig. S6). That enables OGDY/PEO to still maintain good mechanical properties even at high temperatures, which ensures that the battery can operate stably. The stress–strain test shows that OGDY/PEO has better mechanical properties (Fig. 2e) [68]. After the addition of OGDY, the tensile strain rate of the polymer solid electrolyte was increased from 950% to 1280%. DSC showed that the glass transition temperature (T_g_) of PEO was −43.5 °C, while the T_g_ of OGDY/PEO was even lower at −54.9 °C (Fig. 2f). This means that OGDY/PEO has a larger range of amorphous states. Meanwhile, OGDY/PEO has low crystallinity. As shown in XRD, two peaks at 23.2° and 19.0° are observed for PEO and OGDY/PEO (Fig. 2g). The intensity of the diffraction peaks of OGDY/PEO is significantly weaker than that of PEO, which indicates the lower crystallinity of OGDY/PEO [69]. In addition, SEM also proves that OGDY/PEO have lower crystallinity. At similar thicknesses, PEO has obvious grain boundaries (Fig. 2i, j), and its grain diameter is 45–55 nm (Fig. S8). However, the OGDY/PEO surface is flat and smooth, and there is no obvious grain (Fig. 2l, m). To deeply investigate the mechanism by which OGDY influences the crystallization behavior of PEO, we conducted a systematic study using FTIR spectroscopy and DFT calculations. In the FTIR spectra, the vibrational peak near 1142 cm^−1^ is attributed to the stretching vibration of the C–O–C bond in PEO, while the peak near 1346 cm^−1^ corresponds to the stretching vibration of the C–H bond in PEO. The vibrational peak near 3479 cm^−1^ is associated with the stretching vibration of the -OH group in PEO (Fig. S9a–c). After the incorporation of OGDY, the characteristic vibrational peaks of PEO exhibited shifts, indicating the presence of interactions between OGDY and PEO [43]. This interaction is likely attributed to electrostatic attraction between the oxygen atoms with negative electrostatic potential (or hydrogen atoms with positive electrostatic potential) in PEO and the EDR (or ERR) in OGDY (Fig. S9d). Calculations based on the Gaussian B3LYP/6-311G method revealed that the strength of the electrostatic interaction between OGDY and PEO is −1.61 kcal mol^−1^. Additionally, the 2D high-aspect-ratio structure of OGDY imposes significant steric hindrance effects on the ordered arrangement of PEO molecular chains (Fig. 2n, o) [70]. This steric hindrance, combined with the electrostatic interaction, collectively contributes to the reduction in PEO crystallinity. The decreased crystallinity implies enhanced segmental mobility of PEO chains, which creates more favorable conditions for lithium-ion transport. Specifically, the improved chain mobility facilitates the formation of ion-conduction pathways, thereby potentially enhancing the ionic conductivity of the CPSEs. The lower crystallinity indicates that the motility of the PEO chain segments in OGDY/PEO is stronger. The acceleration of polymer chain segment motion provides more favorable conditions for Li^+^ migration, which is expected to improve the ionic conductivity of CPSE [71, 72].

### Electrochemical Performance

**Fig. 3:**
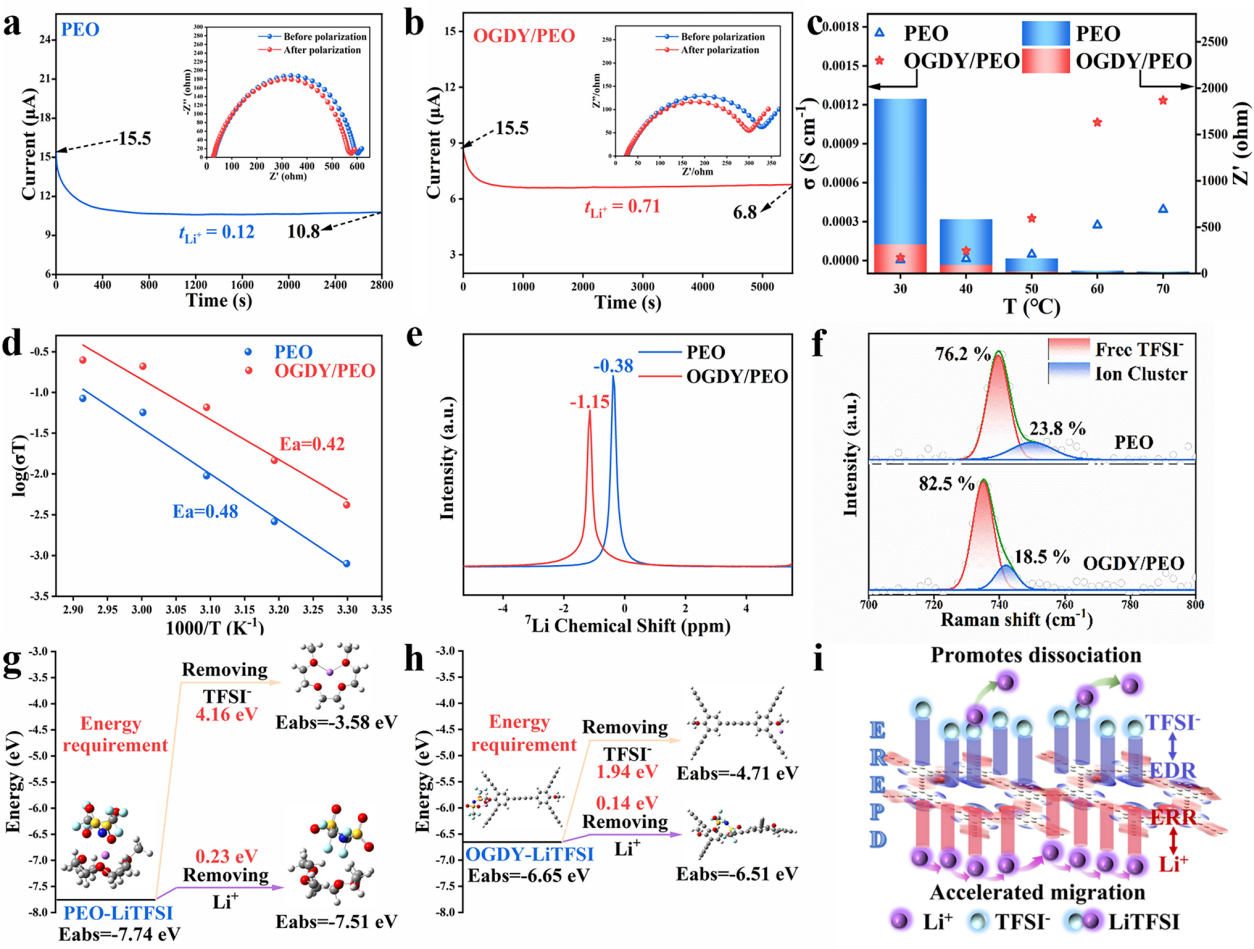
Electrochemical performance of OGDY/PEO. Chronoamperometry curve of **a** Li|PEO|Li and **b** Li|OGDY/PEO|Li symmetric cell under 10 mV polarization. Inset: the EIS before and after potential polarization. **c** Comparison of σ and surface resistance of PEO and OGDY/PEO. **d** Arrhenius plots of ionic conductivities for PEO and OGDY/PEO electrolytes. **e** ^7^Li NMR and **f** Raman spectra of PEO and OGDY/PEO membranes. Step diagram of the energy required by **g** PEO-LiTFSI and **h** OGDY-LiTFSI to produce free ions TFSI^−^, and Li^+^. **i** Schematic diagram of OGDY promoting lithium salt dissociation and Li^+^ migration

We systematically optimized the composite ratio of OGDY (Fig. S10) and subsequently investigated the electrochemical properties of both the optimal OGDY/PEO composite (2 wt%) and pure PEO. The t_Li+_ of two PSEs was systematically investigated using the potentiostatic polarization method. Symmetric cells, Li|PEO|Li and Li|OGDY/PEO|Li, were constructed, and the chronoamperometry curves and EIS before and after polarization were obtained under a constant voltage of 10 mV (Fig. 3a, b). The test results revealed that the t_Li+_ of pure PEO electrolyte was only 0.12, while that of the OGDY/PEO composite electrolyte significantly increased to 0.71, nearly six times higher than that of pure PEO. To further evaluate the ion transport properties of the materials, EIS measurements were conducted on blocking cells, SS|PEO|SS and SS|OGDY/PEO|SS, at different temperatures (30–60 °C), and the σ at the corresponding temperatures was calculated (Fig. S11). As the test temperature increased, the σ of both electrolytes showed an upward trend, while their interfacial resistance significantly decreased (Fig. 3c). Notably, under the same test conditions, the σ of the OGDY/PEO was consistently higher than that of the pure PEO system, and its interfacial resistance was always lower than that of pure PEO (Table S3). At 60 °C, the σ of OGDY/PEO reached 1.1 × 10^–3^ S cm^−1^, an order of magnitude higher than that of pure PEO (2.7 × 10^–4^ S cm^−1^). Compared with similar materials reported in the literature, the OGDY/PEO composite electrolyte demonstrated significant advantages in both σ and t_Li+_ (Table S4). Furthermore, we investigated the ionic conductivity and interfacial impedance of OGDY/PEO under elevated temperatures (80, 100, and 150 °C) (Fig. S12 and Table S5). The results demonstrate that as temperature increases, OGDY/PEO exhibits progressively enhanced ionic conductivity while showing reduced interfacial resistance. Remarkably, at 150 °C, OGDY/PEO achieves an outstanding ionic conductivity of 4.2 × 10⁻^3^ S cm⁻^1^ with a low interfacial resistance of merely 1.98 Ω, indicating excellent high-temperature stability and interfacial compatibility.

In order to further investigate the reason for the high σ and t_Li+_ of OGDY/PEO, the activation energy was calculated based on the classical Arrhenius formula (Fig. 3d). The activation energy of OGDY/PEO was calculated to be 0.42 eV, which is significantly lower than that of PEO (0.48 eV). This implies that the energy barrier to be crossed for Li^+^ transport in OGDY/PEO is smaller and the rapid transfer of Li^+^ is easier. Solid-state nuclear magnetic resonance (NMR) experiments were also carried out (Fig. 3e). Compared with the PEO membranes, the ^7^Li NMR signals of OGDY/PEO were negatively shifted by 0.77 ppm, which indicated that the interactions between the electrolyte membranes and Li^+^ had weakened [73]. This also can contribute to the fast transport of Li^+^ [74]. The degree of dissociation of LiTFSI can be determined using Raman spectroscopy (Fig. 3f) [75]. OGDY/PEO has more TFSI^−^ (82.5%), which is significantly more than 76.2% in PEO, indicating that the introduction of OGDY promotes the efficient dissociation of LiTFSI and effectively inhibits the formation of ionic clusters in the CPSEs. This will help increase the number of free Li^+^.

The above statements are also confirmed by DFT calculation. The binding energies (BEs) of TFSI^−^/Li^+^ in PEO and OGDY were calculated. To ensure the comprehensiveness of the computational results, we selected two configurations (2-configuration and 7-configurations) from the eight configurations of OGDY for modeling (Fig. S13b, g). OGDY has multiple interaction sites with TFSI^−^ and Li^+^, which are labeled as 2-Li-x, 7-Li-x and 2-T-x (x = 1, 2, 3, 4) (Fig. S14). As shown in Fig. S14a, the BEs of PEO-Li^+^ (−4.16 eV) and TFSI-Li^+^ (−6.11 and −5.69 eV) are significantly stronger than those of OGDY-Li^+^ (−1.29 ~ −2.52 eV). This suggests that OGDY results in a lower resistance to Li^+^ shuttling and provides more channels for the fast shuttling of Li^+^. In addition, the binding energies of the 2-configurations OGDY with TSFI^−^ are mostly stronger than those of PEO (Fig. S14b). This result suggests that OGDY has a stronger interaction with TFSI^−^, so that TFSI^−^ can be fixed more effectively. That is favorable for the dissociation of LiTFSI, increasing the concentration of free Li^+^. This statement is also demonstrated by dissociation energy calculations. The energies required for OGDY to produce free TFSI^−^ and Li^+^ were 1.94 and 0.14 eV, respectively (Fig. 3h). These are both smaller than the energies required for PEO to produce free TFSI^−^ and Li^+^ (4.16 and 0.23 eV, respectively, Fig. 3g).

Based on the previously discussed electronic structure of OGDY, including the abundant EDRs, ERRs, and EREPDs, we propose a mechanism for enhanced Li^+^ migration in OGDY/PEO (Fig. 3i). Specifically, ERRs act as Lewis base sites, strongly interacting with Li^+^ and competing with PEO for Li^+^ adsorption. This competition weakens the binding energy between Li^+^ and PEO chains, reduces the activation energy barrier for Li^+^ migration, and facilitates Li^+^ transport within CPSEs [76]. Simultaneously, EDRs function as Lewis acid sites, specifically binding with TFSI^−^ anions. This interaction inhibits the recombination of TFSI^−^ and Li^+^ and promotes the dissociation of lithium salts, increasing the concentration of free Li^+^ in the system and providing more charge carriers for ion transport [77]. Furthermore, the uniformly distributed EREPDs on the large 2D conjugated plane of OGDY, generate a continuous built-in electric field through coupling effects and create a significant potential gradient. This potential gradient provides a low-energy-barrier pathway for the rapid directional migration of Li^+^ [78–80]. This synergistic effect significantly enhances Li^+^ transport efficiency. Owing to these mechanisms, the OGDY/PEO system exhibits excellent σ and a high t_Li+_.

**Fig. 4:**
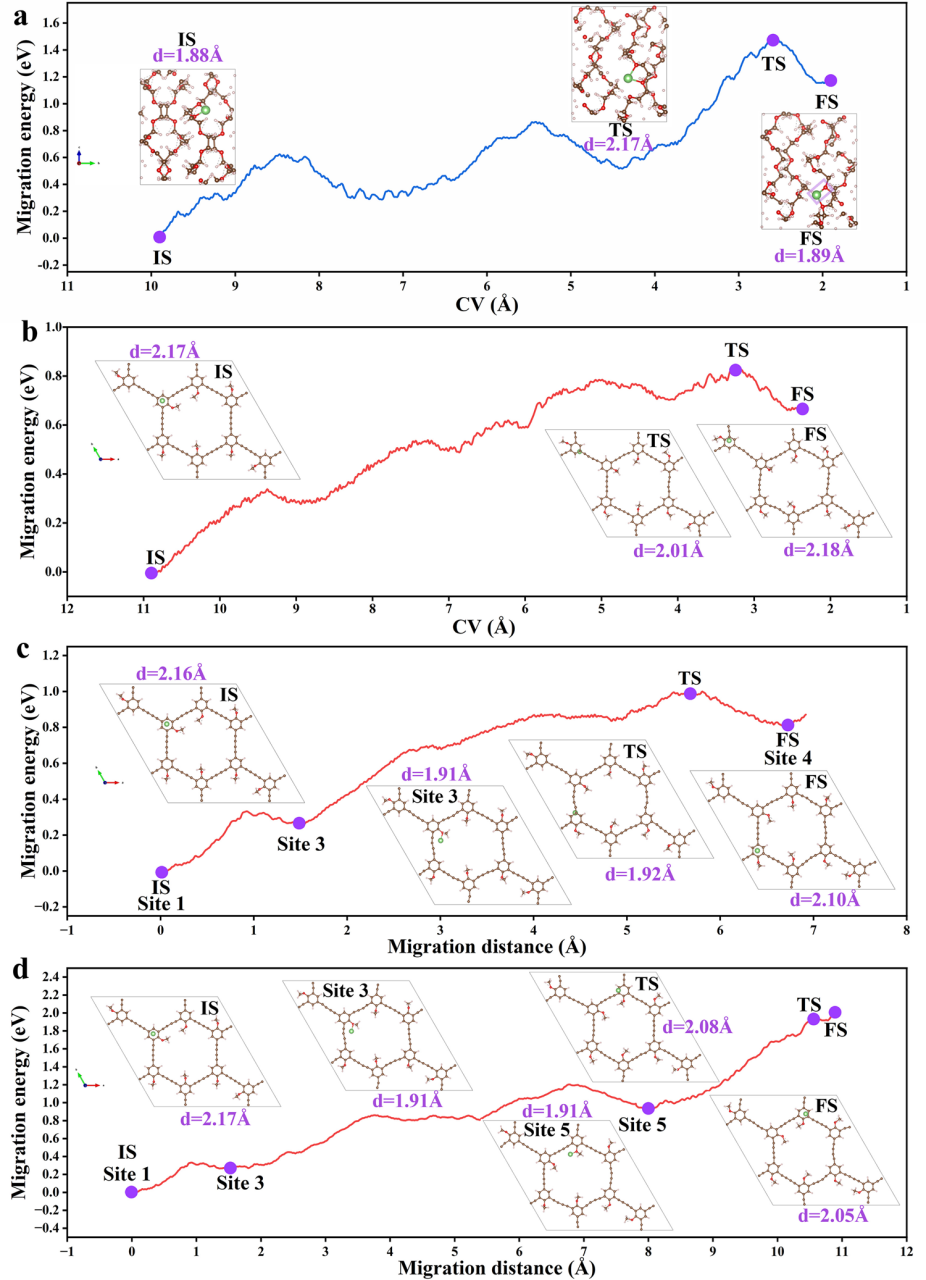
**a** Migration energy barrier of Li^+^ in PEO. **b** Migration pathway of Li^+^ passing through zero methoxy groups on OGDY and the corresponding energy barrier. **c** Migration pathway of Li^+^ passing through one methoxy group on OGDY and the corresponding energy barrier. **d** Migration pathway of Li^+^ passing through two methoxy groups on OGDY and the corresponding energy barrier. The illustrations depict snapshots of Li^+^ migration at critical positions: initial state (IS), transition state (TS), final state (FS), and selected computational nodes (Sites 1, 2, 3, 4), where the distances between Li^+^ and the nearest carbon or oxygen atoms are given

In order to further verify the promoting effect of OGDY on Li^+^ migration, the migration paths and energy barriers (ER) of Li^+^ in pure PEO and OGDY systems were calculated by AIMD and Slow-growth approach simulations calculations (Fig. 4). The results demonstrate that Li^+^ migration in PEO requires a significantly high energy barrier of 1.48 eV (Fig. 4a), whereas in OGDY, the energy barrier dramatically decreases to only 0.83 eV (Fig. 4b). This directly confirms OGDY's ability to facilitate Li^+^ migration and validates the effectiveness of our EREPD strategy. Furthermore, we systematically compared the energy barriers for Li^+^ migration along three distinct pathways containing different numbers of -OMe functional groups: E_R2_ (two -OMe groups), E_R1_ (one -OMe group), and E_R0_ (zero -OMe groups) (Fig. 4b–d). The calculated energy barriers follow the trend E_R2_ (1.92 eV) > E_R1_ (0.99 eV) > E_R0_ (0.83 eV). Combined with the videos of AIMD simulation (see Movie S1–S4 in the Web enhanced object for motion), it was found that Li^+^ migration on OGDY primarily occurs along the *sp*^2^ carbons of the benzene ring and the sp carbons of the diacetylene bonds. Furthermore, based on the analysis of the minimum Li^+^-OGDY distances during migration, the oxygen sites in the OGDY/PEO system demonstrate the strongest Li^+^ binding affinity, which explains the high barrier of the Li^+^ migration between the two -OMe functional groups on OGDY. Notably, when Li^+^ migrates near the O atoms, its closest distance to OGDY (~ 1.91 Å) remains in the upper limit of the corresponding value in the PEO system (1.88 ~ 2.17 Å). This observation suggests that even when OGDY exhibits strong interaction with Li^+^, it is still weaker compared to the PEO system. This finding further confirms that OGDY can promote Li^+^ migration through a competitive adsorption effect.

### Performance of Li||Li Symmetrical Cells

**Fig. 5:**
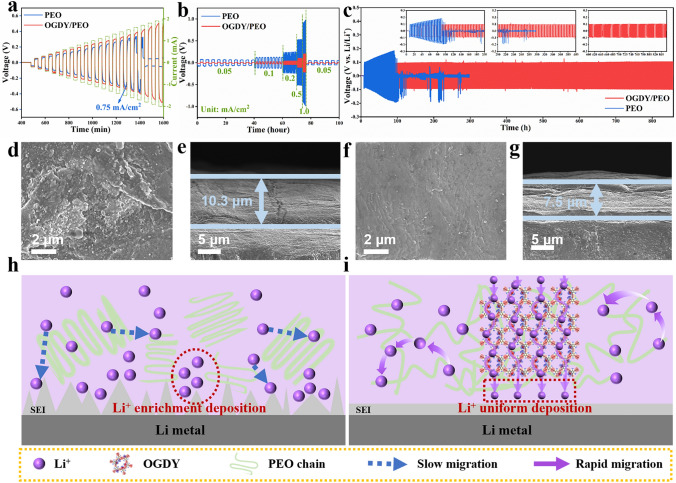
**a** Critical current density tests of Li|PEO|Li and Li|OGDY/PEO|Li symmetric batteries. **b** Galvanostatic cycling of the symmetric cells at different current densities. **c** Long-term cycle performance of Li|PEO|Li and Li|OGDY/PEO|Li symmetric cells at 60 °C, 0.1 mA cm^−2^, and 0.1 mAh cm^−2^. SEM images of Li metal anode surface after 60 cycles in **d** Li|PEO|Li and **f** Li|OGDY/PEO|Li symmetric cells. Cross-sectional view of the SEI layer on the lithium anode surface in **e** Li|PEO|Li and **g** Li|OGDY/PEO|Li symmetric cells after 60 cycles. Schematic of SEI layer formation in **h** Li|PEO|Li and **i** Li|OGDY/PEO|Li symmetric cells

To reveal the interfacial stability between the PSEs and Li metal anode, the symmetric cells Li|PEO|Li and Li|OGDY/PEO|Li were assembled. The critical current density was tested by stepwise constant-current charging and discharging method from 0.1 to 1.0 mA cm^−2^ (Fig. 5a). The limiting current density of the PEO electrolyte was 0.75 mA cm^−2^. However, the OGDY/PEO electrolyte can be stably cycled at current densities over 1 mA cm^−2^ without overpotential mutations. In addition, the symmetric cells Li|PEO|Li and Li|OGDY/PEO|Li were cycled at current densities ranging from 0.05 to 1 mA cm^−2^ at 60 °C, respectively. As shown in Fig. 5b, the Li|OGDY/PEO|Li cell has a smaller overpotential than Li|PEO|Li at each current density. This indicates that the interface resistance between the OGDY/PEO film and the lithium metal electrode is lower. This is consistent with the results from the previous electrochemical tests (Fig. 3c). The Li|OGDY/PEO|Li cell has a good and long cycle life, and it can be operated stably for 850 h at 60 °C, 0.1 mA cm^−2^, and 0.1 mAh cm^−2^ (Fig. 5c). In contrast, the Li|PEO|Li cell had increasing voltage with the increasing number of cycles, and a short circuit occurred at 90 h with a sudden voltage drop.

To figure out the reason for the long cycle life of Li|OGDY/PEO|Li, the surface of lithium anode in Li|OGDY/PEO|Li and Li|PEO|Li batteries after cycling was characterized by SEM. As shown in Fig. 5d, a large number of lithium dendrites were generated on the surface of the lithium anode in the Li|PEO|Li after 60 cycles, and the surface was very rough. Whereas, the Li|OGDY/PEO|Li lithium anode surface retained its original flat and smooth morphology after 60 cycles, and no lithium dendrites were generated (Fig. 5f). In addition, it is worth noting that the SEI layer produced on the Li|PEO|Li lithium anode surface is 10.3 μm, while the SEI layer on the Li|OGDY/PEO|Li lithium anode surface is only 7.5 μm (Fig. 5e, g). The thinner SEI layer implies less active lithium loss, which helps mitigate irreversible capacity degradation of the battery [81]. Meanwhile, the uniform and thin SEI layer contributes to reduced interfacial resistance [82, 83], minimized mechanical fractures caused by volume changes [84], optimized interfacial contact, and suppressed interfacial side reactions [85]. The dendrite-suppressing effect of OGDY/PEO, synergistically combined with the thin SEI layer, collectively ensures long-term stability at the electrode/CPSEs interface, thereby significantly improving the battery's cycling performance.

To elucidate the reason for the increased stability of the OGDY/PEO interface, the chemical composition of the SEI layer was analyzed by XPS (Fig. S15). The results show that the contents of inorganic lithium species LiF, Li_3_N, and Li_2_O are comparable in the SEI layer on the lithium anode surface of Li|PEO|Li and Li|OGDY/PEO|Li. This reminds us that the generation of inorganic lithium species is not the main reason for the formation of a uniform and dense SEI layer in Li|OGDY/PEO|Li. Combined with the previous discussion, we deduce that the promotion of Li^+^ migration by OGDY is the main reason for the inhibition of lithium dendrite growth [86]. The specific mechanism is shown in Fig. 5h, i. In the PEO system, the migration of Li^+^ is more difficult due to the high crystallinity of PEO. That leads to the uneven distribution of local Li^+^ concentration. Li^+^ will be preferentially deposited at the raised sites at the interface between PEO and electrode, forming lithium dendrites. In the OGDY/PEO system, the introduction of OGDY significantly reduces the crystallinity of the PEO while enhancing the mobility of polymer segments. This structural feature synergizes with the Lewis acid–base pair interactions induced by EDRs and ERRs in OGDY, collectively promoting the rapid migration of Li^+^ between polymer segments. That effectively mitigates concentration polarization at the electrode/electrolyte interface, preventing uneven lithium deposition caused by Li^+^ distribution inhomogeneity. Furthermore, the uniformly distributed EREPDs on the 2D plane of OGDY provide directional migration channels for Li^+^, facilitating the uniform nucleation and growth of lithium metal. This uniform Li^+^ distribution effectively suppresses the localization of current density, thereby significantly reducing the growth of lithium dendrites [87].

### Full Cells Performance

**Fig. 6:**
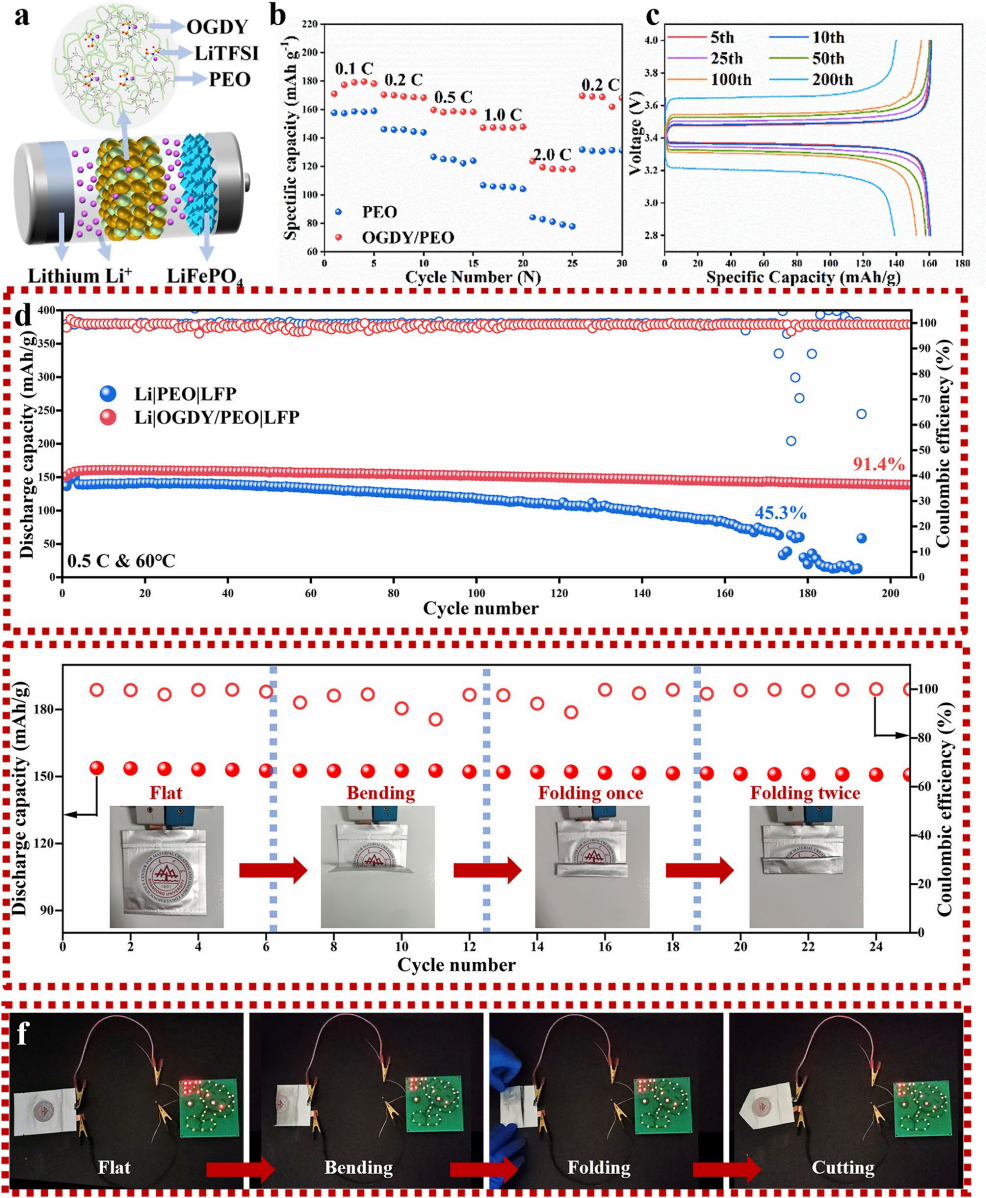
Performance of Li||LFP full cells. **a** Schematic diagram of the full battery structure. **b** Rate performance of Li|OGDY/PEO|LFP and Li|PEO|LFP full cell at 0.1 C, 0.2 C, 0.5 C, 1.0 C, and 2.0 C. **c** Charge/discharge voltage profiles Li|OGDY/PEO|LFP at 0.5 C. **d** Cycling performance of the Li|OGDY/PEO|LFP full cell at 0.5 C. **e** Cycling stability of Li|OGDY/PEO|LFP pouch cell. **f** Pictures of Li|OGDY/PEO|LFP pouch cell lighting LED lights at room temperature in different states

In order to investigate the effect of OGDY composite on the battery performance, performance tests of all-solid-state lithium metal batteries were conducted. The LSV tests revealed that OGDY/PEO has a wider range of electrochemical stability, which provides greater flexibility in battery design (Fig. S16). Here we selected LFP as the cathode material and Li metal as the anode to assemble LFP|OGDY/PEO|Li and LFP|PEO|Li full cells (Fig. 6a). As shown in Fig. 6b, Li|OGDY/PEO|LFP has excellent rate performance. The discharge-specific capacity of Li|OGDY/PEO|LFP at 60 °C with 0.1 C, 0.2 C, 0.5 C, and 1.0 C are 179.1, 169.2, 158.7, and 147.3 mAh g^−1^, respectively. When the current density reaches a relatively high level of 2.0 C, the discharge-specific capacity of the battery can still be maintained at 118.2 mAh g^−1^. This means that Li|OGDY/PEO|LFP has excellent energy storage capacity even under high current charging and discharging conditions. Conspicuously, the discharge-specific capacity of the Li|OGDY/PEO|LFP is consistently higher than that of the Li|PEO|LFP. Moreover, when the current density is restored to 0.2 C, the discharge-specific capacity of Li|OGDY/PEO|LFP quickly returns to 168.8 mAh g^−1^, with the capacity recovery approaching 100%. That indicates that Li|OGDY/PEO|LFP has good cycling stability under different multiplication rates. Li|OGDY/PEO|LFP full cell was activated for 3 cycles at 60 °C with 1.0 C, and then cycled at 60 °C with 0.5 C to prove its stability (Fig. 6d). The Li|OGDY/PEO|LFP was able to operate stably for more than 200 cycles without short circuits and a coulombic efficiency of 91.4%. Its initial specific capacity is 159.3 mAh g^−1^. The cycling performance of Li|OGDY/PEO|LFP is significantly better than that of Li|PEO|LFP. Under the same conditions, the Li|PEO|LFP battery had a short circuit in 173 cycles, and its specific capacity rapidly attenuated from the initial 139.4 to 63.1 mAh g^−1^, with an extremely low coulombic efficiency of 45.3%. In addition, through the charge/discharge voltage profiles during the cycling process, we found that the initial polarization voltage of Li|OGDY/PEO|LFP cells is 114 mV, which is much smaller than the initial polarization voltage of Li|PEO|LFP (421 mV) (Figs. 6c and S17). This is mainly due to the fact that OGDY/PEO has a smaller charge migration resistance and higher σ than PEO. Meanwhile, the polarization voltage of OGDY/PEO changed less and only increased by 134 mV when cycling for 100 cycles. However, the polarization voltage of PEO increased dramatically to 1000 mV and the polarization voltage plateau fluctuated significantly after cycling for 100 cycles. These results again indicate that the Li|OGDY/PEO|LFP has superior cycling stability.

To further demonstrate the excellent mechanical properties, high safety, and practical application value of OGDY/PEO CPSE, we also assembled Li|OGDY/PEO|LFP pouch cells. The Li|OGDY/PEO|LFP pouch cell can be stably cycled at 60 °C. It can achieve a specific capacity of 154 mAh g^−1^ at 0.1C even after bending and multiple folds (Fig. 6e). At room temperature, this pouch cell can light up a flower pattern of 42 red and yellow light-emitting diode (LED) lamps (Fig. 6f). When the pouch battery was bent, folded, and cut, the battery did not experience short circuit or thermal runaway, and could still maintain the stable operation of LED lamps. These results show that the Li|OGDY/PEO|LFP pouch battery has good flexibility, mechanical properties, and safety. It can meet the demands of complex practical applications.

The EREPD induced by asymmetric methoxy substitution significantly improves the performance of OGDY/PEO CPSEs, including σ, t_Li+_, and battery cycling stability. To systematically validate the effectiveness of this asymmetric substitution strategy, we further designed and prepared symmetrically hydrogen-substituted GDY (HGDY) as a comparative filler (Fig. S18), constructing the HGDY/PEO CPSEs. Experimental results demonstrate that HGDY/PEO exhibits a σ of only 3.8 × 10^–4^ S cm^−1^ and a t_Li+_ of 0.65 at 60 °C, significantly lower than those of OGDY/PEO (*σ* = 1.1 × 10^–3^ S cm^−1^, t_Li+_ = 0.71) (Fig. S19). This distinct difference directly confirms the critical role of asymmetric substitution groups (-OCH_3_) in facilitating directional Li^+^ transport. In symmetric cell tests, although Li|HGDY/PEO|Li shows improved cycling stability (220 h) compared to the pure PEO system (90 h), it falls far short of the long-term cycling performance of Li|OGDY/PEO|Li (850 h) (Fig. S20). Full-cell tests further reveal the performance gap between the two material designs: The Li|HGDY/PEO|LFP battery exhibits capacity retention of only 65.3% after 173 cycles, which sharply declines to 46.1% after 200 cycles, whereas Li|OGDY/PEO|LFP maintains 91.4% capacity retention even after 205 cycles (Fig. S21). These comparative experiments clearly demonstrate the unique advantages of the asymmetric substitution strategy: By introducing the EREPD effect, Li⁺ transport kinetics can be effectively optimized, leading to comprehensive performance enhancement of solid-state electrolytes.

## Conclusions

In summary, the OGDY prepared by the asymmetric substitution strategy not only maintains the order and crystallinity of the 2D structure of GDY but also effectively regulates the charge distribution of OGDY, which leads to a uniform distribution of EREPDs in the 2D plane of OGDY. The Lewis acid–base interactions between the EDR/ERR and the TFSI^−^/Li^+^ facilitated the dissociation of lithium salts and increased the concentration of Li^+^. It also attenuates the interaction between Li^+^ and PEO, which promotes the migration of Li^+^. The increase of σ (1.1 × 10^–3^ S cm^−1^) and t_Li+_ (0.71) in OGDY/PEO promotes the formation of a dense and rigid SEI layer, which helps to inhibit the growth of lithium dendrites and improves the stability of the battery. Li|OGDY/PEO|LFP full cell has a maximum capacity of 158.7 mAh g^−1^ and it can be stably cycled for more than 200 cycles with a capacity retention rate of 91.4% at 0.5 C. The Li|OGDY/PEO|LFP pouch cells provide excellent safety and flexibility, and can stabilize the output voltage to light up LEDs. This work demonstrates that modulating the charge distribution of 2D nanofillers is an effective strategy for improving the performance of PSEs. Future studies could further explore the systematic modulation of the EREPD of 2D nanofillers, including but not limited to the modulation of the spatial distribution of ERRs and EDRs and the precise modulation of the potential gradient. This EREPD-based regulation strategy is expected to further enhance the ion migration property in CPSEs, offering a novel approach to further enhance solid-state battery performance.

## Supplementary Information

Below is the link to the electronic supplementary material.

Supplementary file1 (MP4 1228 kb)

Supplementary file2 (MP4 520 kb)

Supplementary file3 (MP4 515 kb)

Supplementary file4 (MP4 614 kb)

Supplementary file5 (DOCX 7736 kb)
