# Supplementary material for: Enhanced Regional Electric Potential Difference of Graphdiyne Through Asymmetric Substitution Strategy Boosts Li+ Migration in Composite Polymer Solid-State Electrolyte
Source: Nanomicro Lett. 2025 May 21;17:267. doi: 10.1007/s40820-025-01790-5 (PMC12095840; doi:10.1007/s40820-025-01790-5)
Supplement: Supplementary file 5 — Supplementary file5 (DOCX 7736 kb) [file 40820_2025_1790_MOESM5_ESM.docx]

Supporting Information for

**Enhanced Regional Electric Potential Difference of Graphdiyne through Asymmetric Substitution Strategy Boosts Li^+^ Migration in Composite Polymer Solid-State Electrolyte**

Chao Jiang^1♯^, Kaihang Wang^1♯^, Luwei Zhang^1^, Chunfang Zhang^2^*, Ning Wang^1^*

^1^ Shandong Provincial Key Laboratory for Science of Material Creation and Energy Conversion, Science Center for Material Creation and Energy Conversion, School of Chemistry and Chemical Engineering, Shandong University, Jinan 250100, P. R. China

^2^ College of Chemistry and Materials Science, Hebei Key Laboratory of Analytical Science and Technology, Hebei University, Baoding 071002, P. R. China

^♯^Chao Jiang and Kaihang Wang contributed equally to this work.

* Corresponding authors. E-mail: [wang_ning@sdu.edu.cn](mailto:wang_ning@sdu.edu.cn) (Ning Wang); [zhangcf@hbu.edu.cn](mailto:zhangcf@hbu.edu.cn) (Chunfang Zhang)

**Supplementary Figures and Tables**


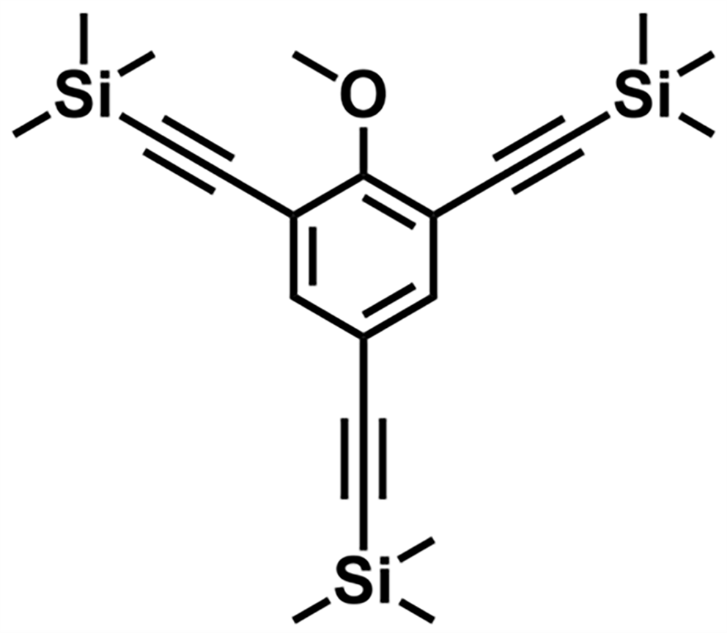


**Fig. S1** The structure of OGDY monomer


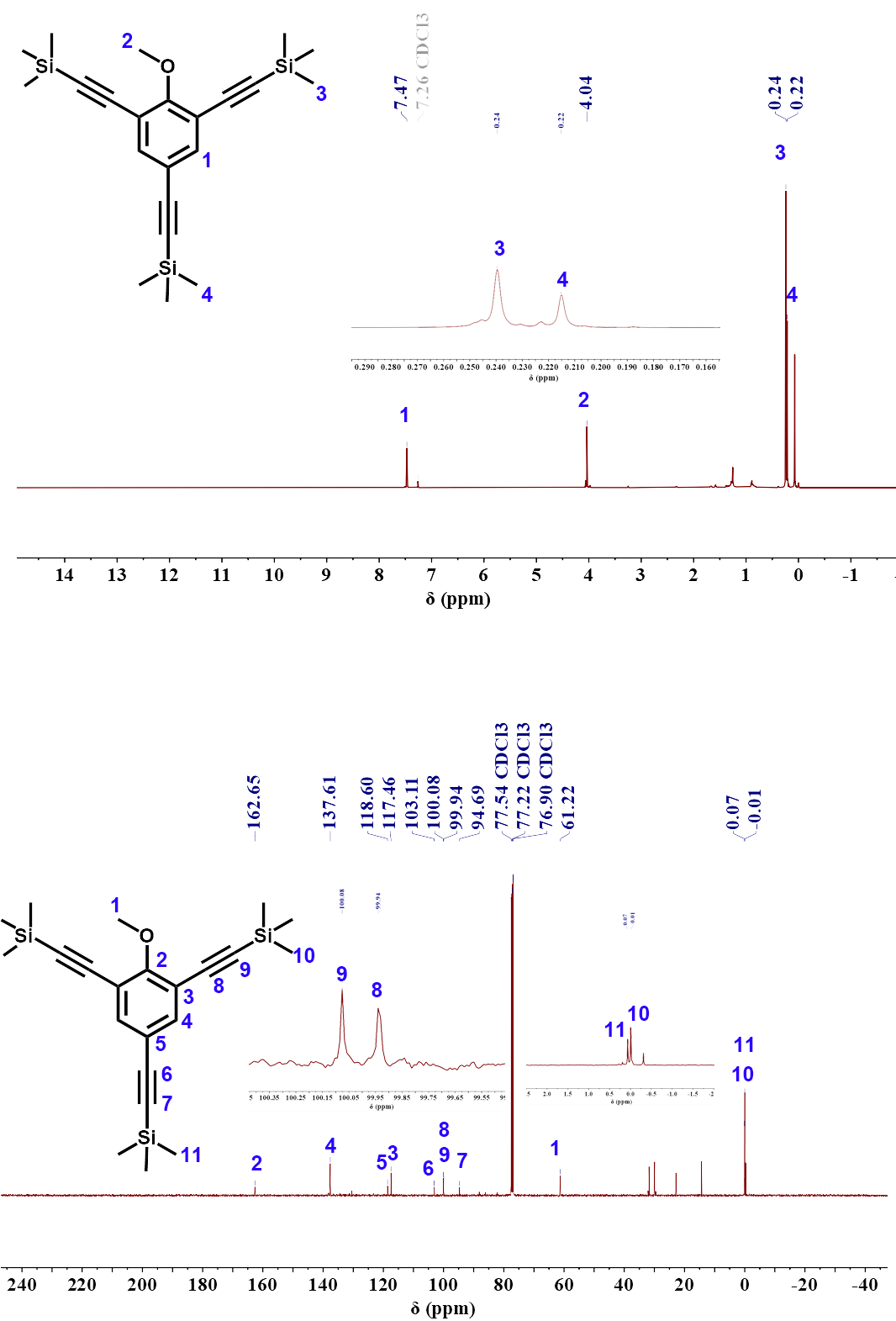


**Fig. S2** The ^1^H NMR spectroscopy of OGDY precursor


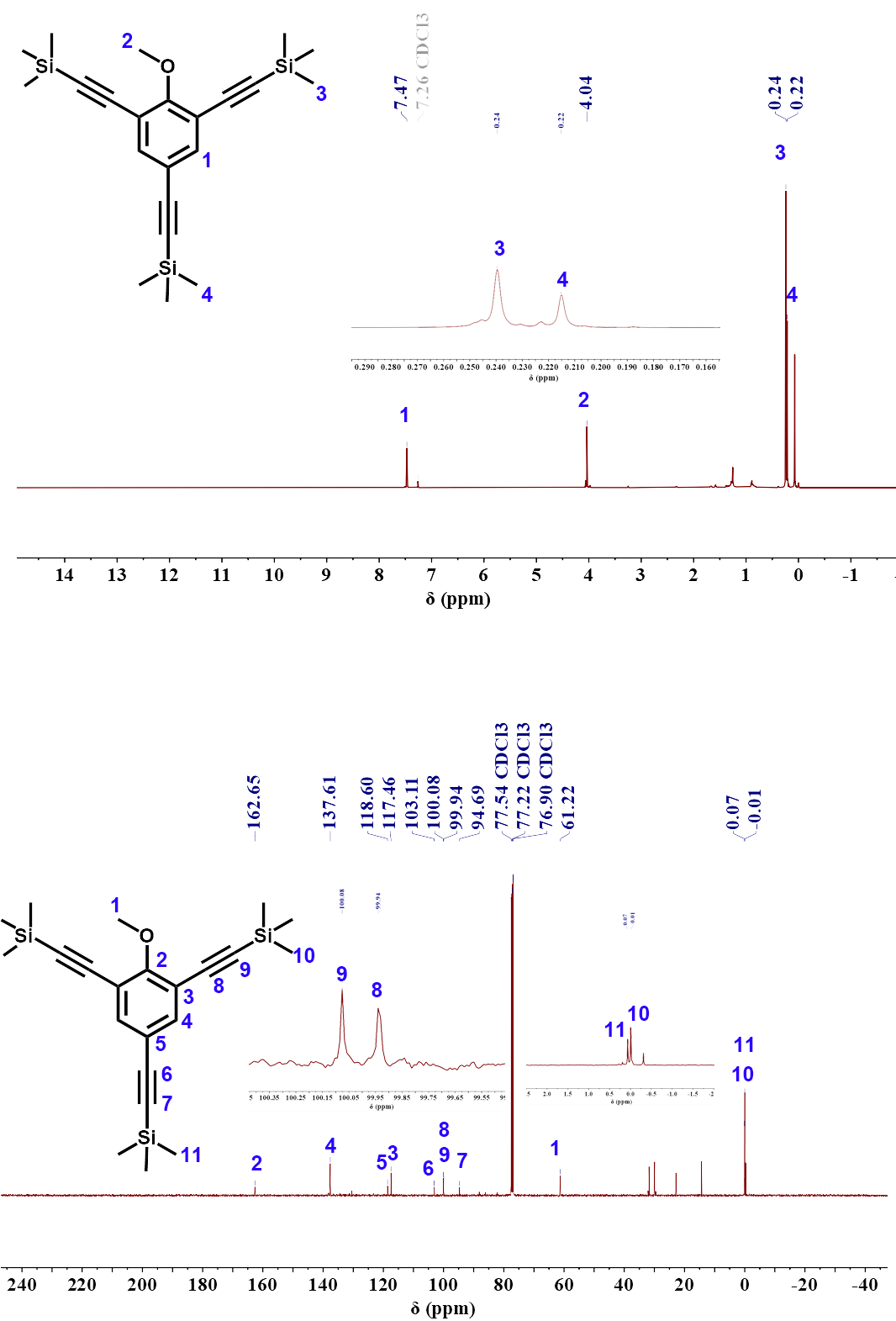


**Fig. S3** The ^13^C NMR spectroscopy of OGDY precursor


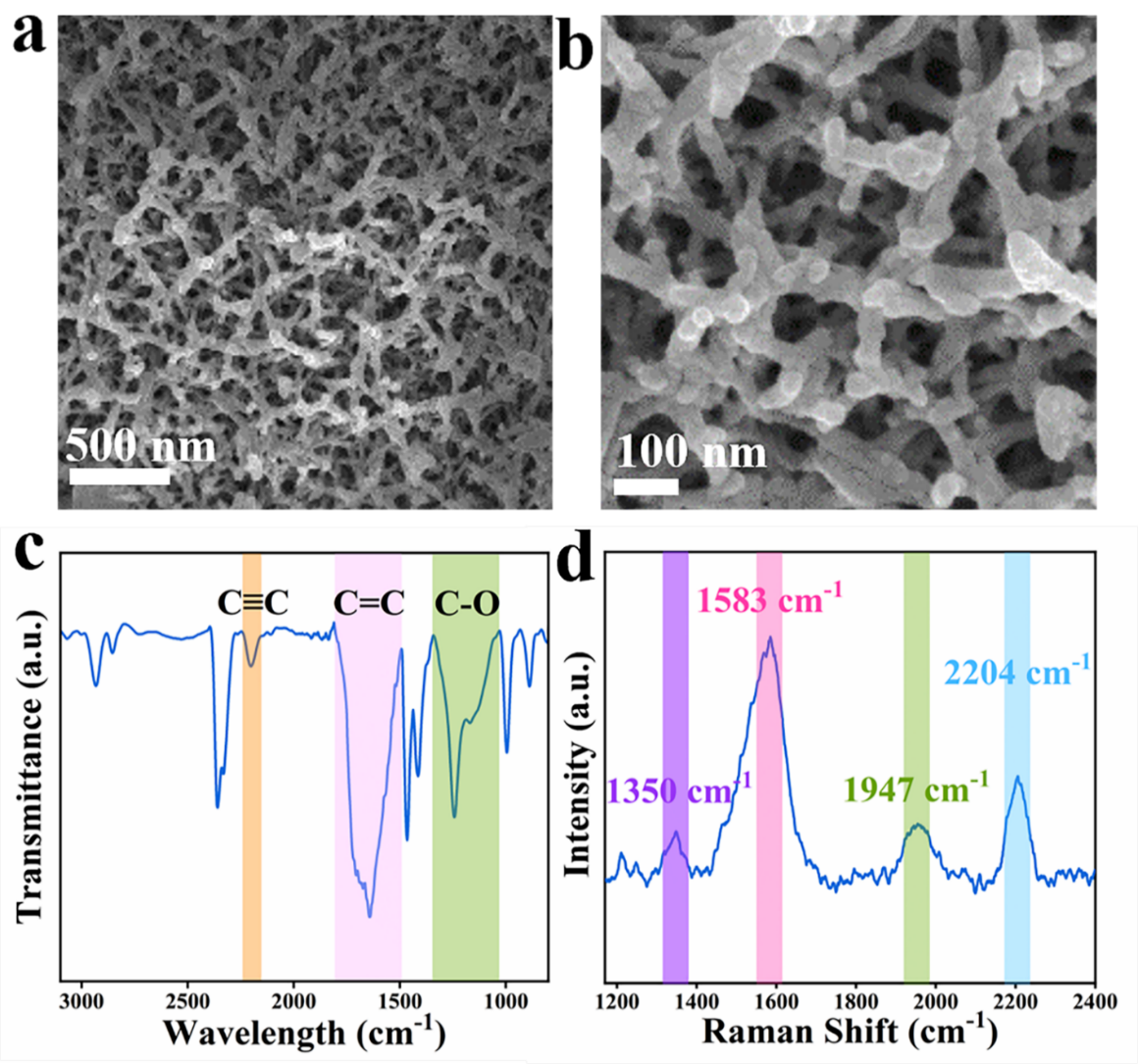


**Fig. S4** (**a, b**) the SEM, (**c**) FTIR, and (**d**) the Raman of OGDY

The top-view SEM suggested that OGDY has a porous network structure composed of nanowires with a diameter of 40 nm (Fig. S4a and b). In the FTIR image of OGDY, the peak at 2150-2250 cm^-1^ is attributed to the stretching vibration of the C≡C (Fig. S4c). The signal between 1500-1800 cm^-1^ is caused by the skeletal vibrations of the benzene ring. The existence of the stretching vibration peak (1000-1300 cm^-1^) of the C-O bond in the methoxy group proves the successful substitution of the methoxy group. In the Raman spectrum of OGDY, the respiratory vibration peak of sp^2^ C is present at 1350 cm^-1^ (Fig. S4d). At the same time, the characteristic peak of in-plane stretching vibration of the aromatic ring appears at 1583 cm^-1^. These two peaks are typical D band and G band of GDY. In addition, the two peaks at 2204 cm^-1^ and 1947 cm^-1^ are attributed to the C≡C stretching vibration.





**Fig. S5** The XRD of OGDY

The X-ray diffraction (XRD) pattern shows one broad characteristic diffraction peak at 20.9° (Fig. S5), which represents a hallmark feature of nanocrystalline materials.


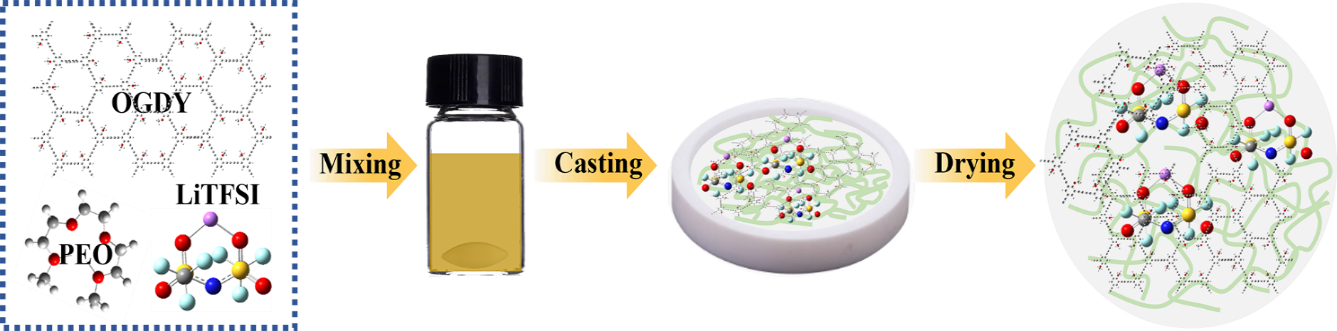


**Fig. S6** Schematic representation of the preparation method of OGDY/PEO CPSE


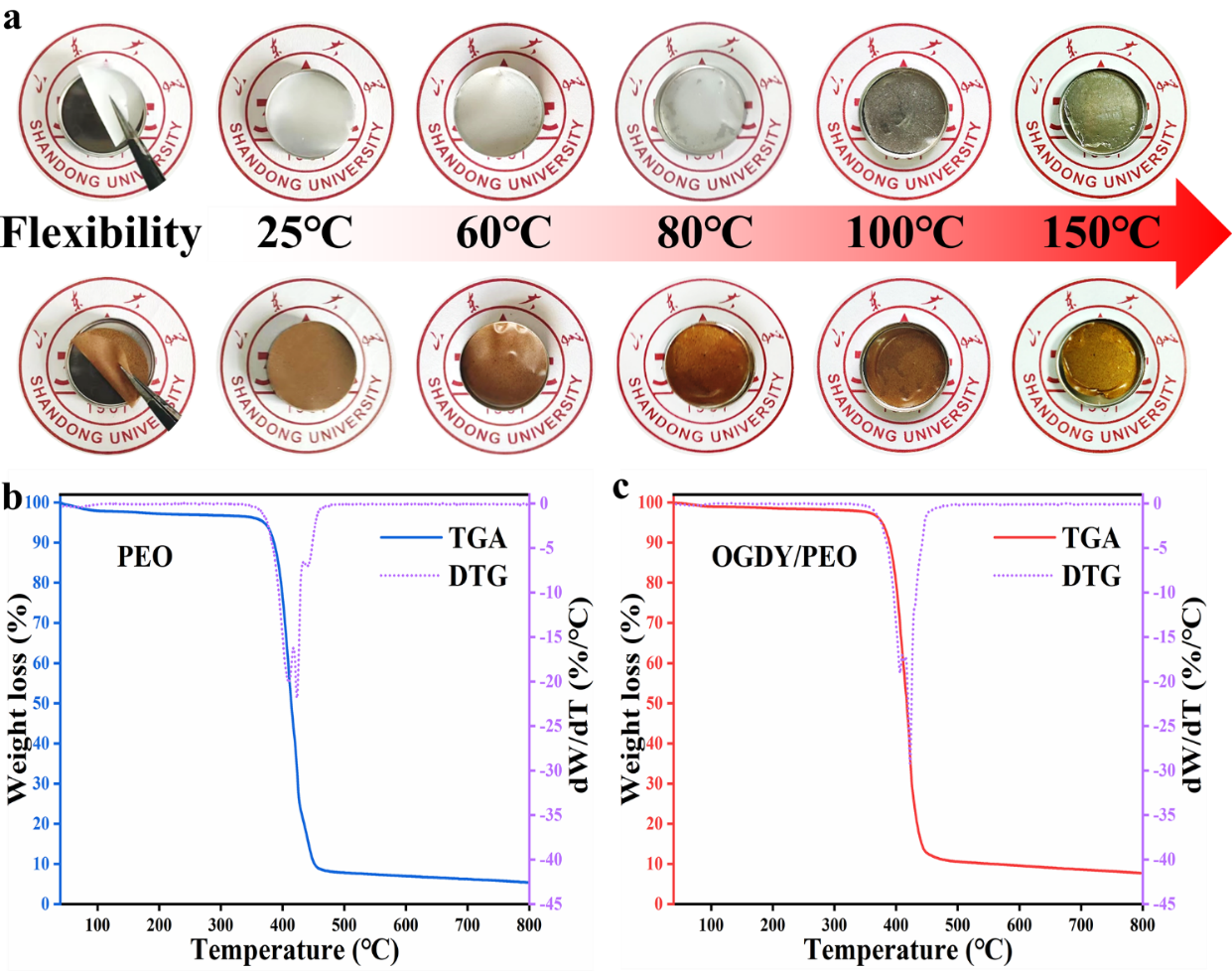


**Fig. S7** (**a**) Optical photograph of PEO (the first line) and OGDY/PEO (the second line) polymer electrolyte at different temperatures. TGA of (**b**) PEO and (**c**) OGDY/PEO polymer electrolyte

To further investigate the thermal stability, OGDY/PEO and PEO were kept in the oven at 25 °C, 60 °C, 100 °C, and 150 °C for 1h (Fig. S7a). As the temperature increases, the solid electrolyte film becomes softer and stickier, which will help to form good interfacial contact between the solid electrolyte film and electrodes. When the temperature reaches 100 °C, the PEO film is near a molten state, which may lead to a short circuit of the battery [1]. In contrast, OGDY/PEO film can still maintain its original form even when the temperature reaches 150 °C. This shows that OGDY/PEO can still maintain good mechanical properties even at high temperatures, which ensures that the battery can operate stably. In addition, TGA is also used to study the thermal stability of these solid electrolyte films (Fig S7b, c). The decomposition temperature of OGDY/PEO (355 °C) is slightly higher than that of PEO (349 °C), which is much higher than the boiling point of liquid electrolyte (120 °C) and the melting point of Li (180 °C). The higher decomposition temperature of OGDY/PEO enables it to fully meet the requirements of thermal stability of solid electrolyte film in practical Li battery applications.





**Fig. S8** Grain size distribution map of PEO polymer electrolyte


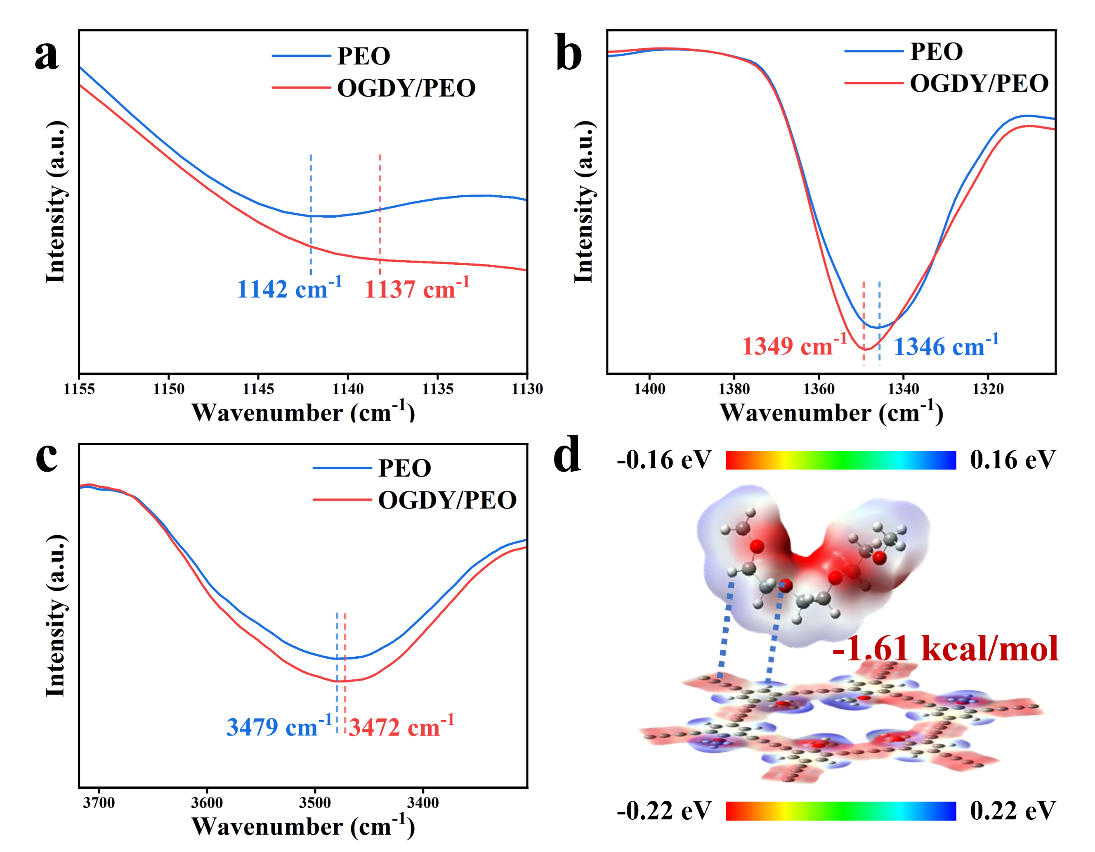


**Fig. S9** (**a-c**) The FTIR of PEO and OGDY/PEO. (**d**) The strength of the electrostatic interaction between OGDY and PEO as calculated by the Gaussian B3LYP/6-311G method


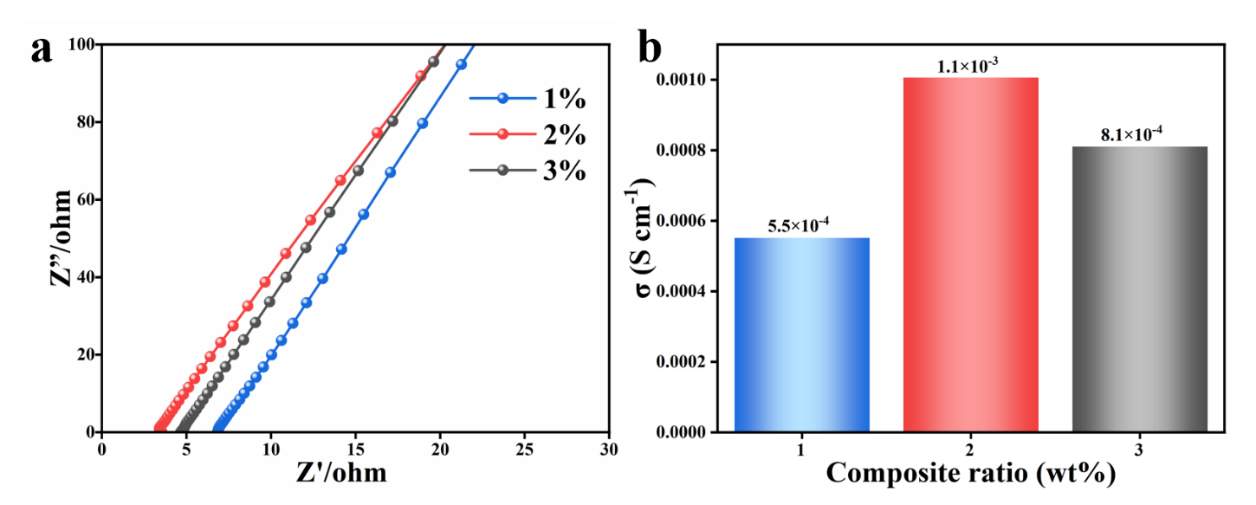


**Fig. S10** (**a**) EIS of different composite ratios OGDY/PEO blocked cells at 60℃. (**b**) σ of different composite ratios OGDY/PEO

OGDY/PEO composites with three different composite ratios (1 wt%, 2 wt%, and 3 wt%) were prepared and assembled into SS||SS blocked cells for EIS measurements at 60 °C. The calculated results revealed that the 2 wt% OGDY/PEO composite exhibited the highest ionic conductivity of 1.01 × 10^-3^ S cm^-1^. Consequently, the optimal composite ratios for OGDY/PEO was determined to be 2 wt%. Notably, a relatively low OGDY loading significantly improved the ionic conductivity of the composite, consistent with the widely reported optimal low-filler-ratio phenomenon in composite polymer electrolytes [S2, S3]. This result further demonstrates the effectiveness of incorporating OGDY into PEO for enhancing the performance. As shown in Fig. S8, when the composite ratio of OGDY increased to 3 wt%, the ionic conductivity decreased. This phenomenon may be attributed to nanoparticle aggregation at higher filler concentrations, which disrupts the continuity and uniformity of ion transport pathways [S4].


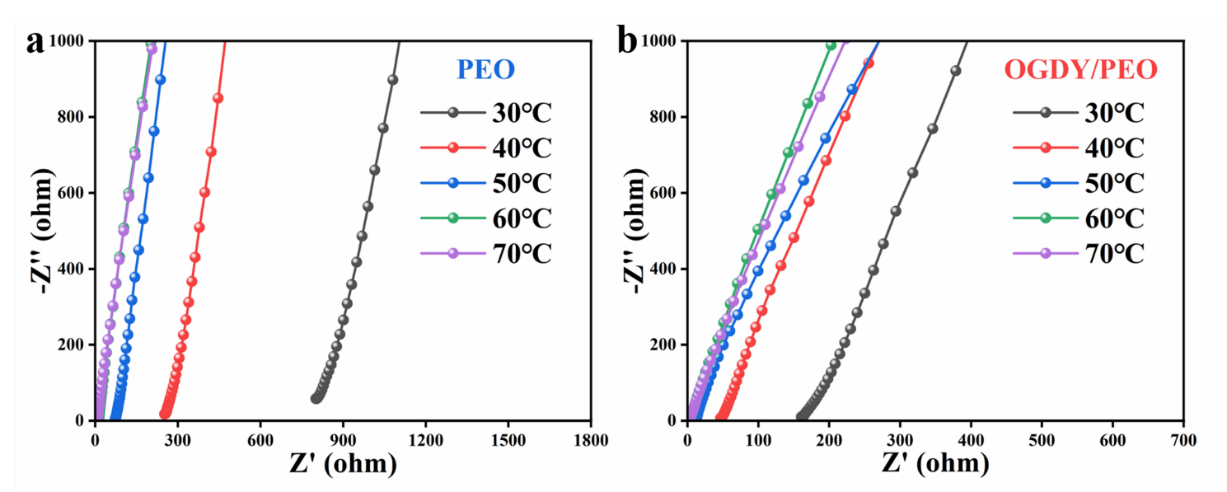


**Fig. S11** EIS of SS|PEO|SS and SS|OGDY/PEO|SS blocked cells at 30℃~70℃


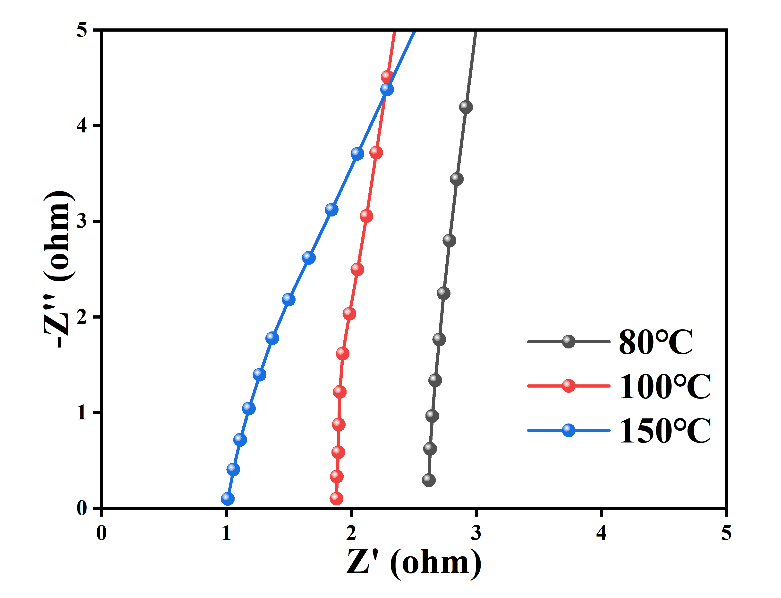


**Fig. S12** EIS of SS|OGDY/PEO|SS blocked cells at 80 ℃, 100 ℃, and 150 ℃


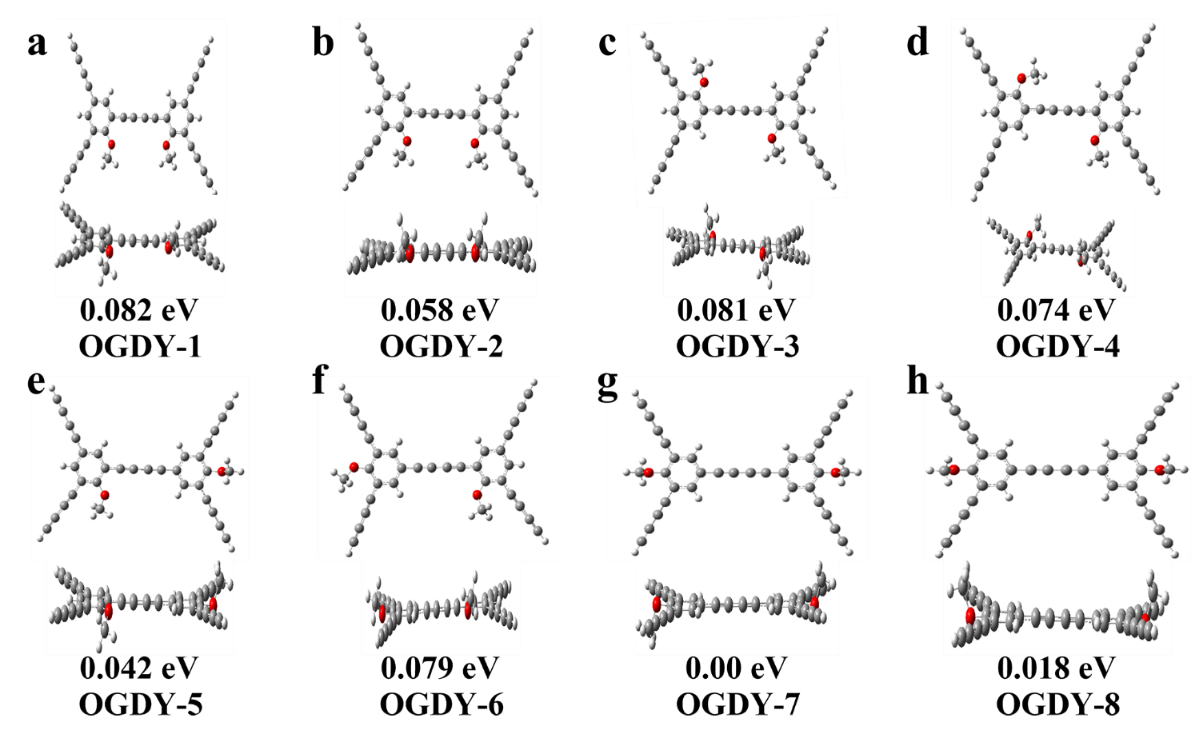


**Fig. S13** The binding energy of eight OGDY configurations calculated by DFT

We have identified 8 basic conformations of OGDY using the connected two monomers precursors as the basic model (Fig. S10). The stabilities of these 8 conformations were calculated by DFT, among which the conformer OGDY-7 has the lowest energy and is the most stable conformer (Fig. S10g). Simultaneously, we found that the interaction pattern between 2-configuration and Li^+^ exhibits similarity to that of PEO (Fig. S10b), where both can form chelation structures with Li^+^ through two oxygen atoms. This coordination mode may significantly enhance the binding energy between OGDY and Li^+^. Therefore, to ensure the comprehensiveness of the computational results, we modeled the BEs of TFSI^-^ and Li^+^ in OGDY with the 2-configuration and 7-configurations, respectively.


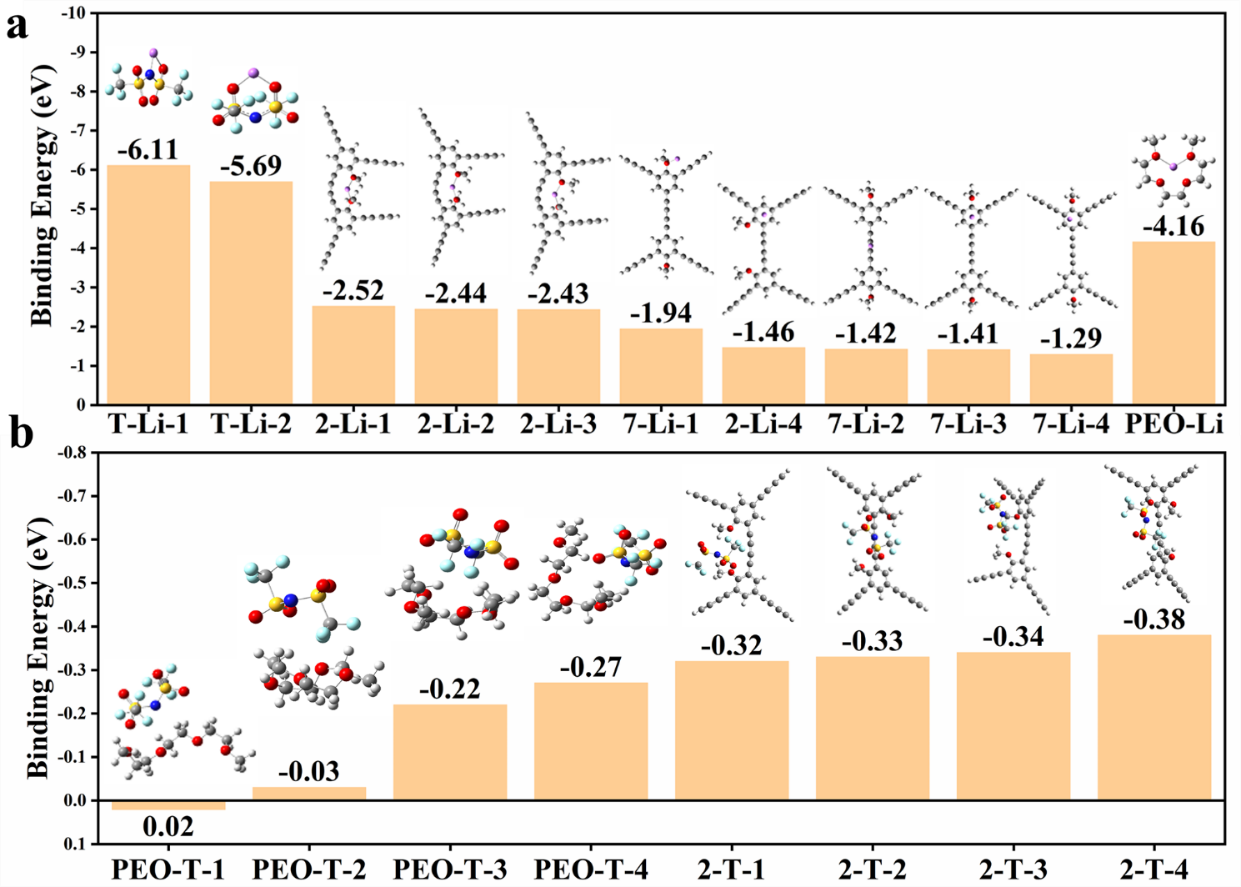


**Fig. S14** DFT calculations. **a**) Binding energies of TFSI-Li^+^, PEO-Li^+^, and OGDY-Li^+^ at different position. **b**) Binding energies of PEO-TFSI^-^, and OGDY-TFSI^-^ at different positions.


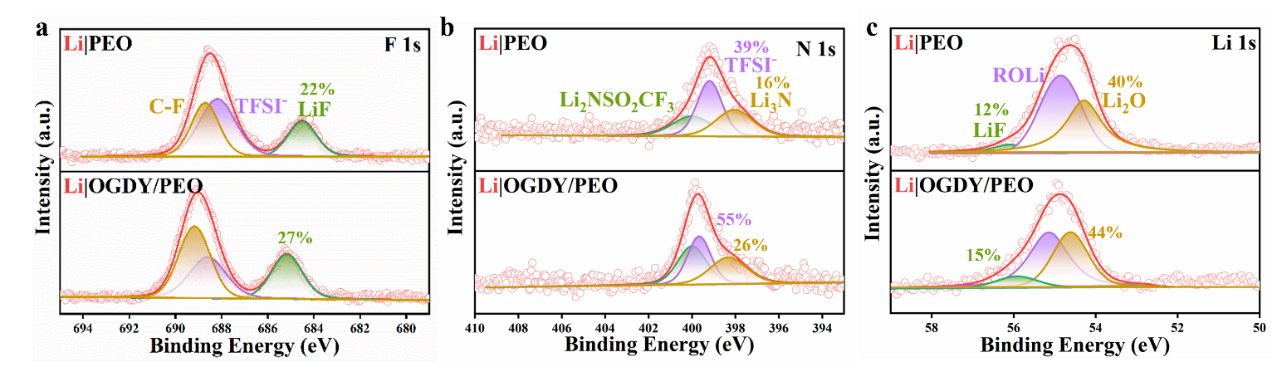


**Fig. S15** XPS spectra of (**a**) F 1s, (**b**) N 1s, (**c**) Li 1s on the surface of lithium anode in batteries after 60 cycles

The results show that both Li|PEO|Li and Li|OGDY/PEO|Li SEI layers formed on the lithium anode surface contain inorganic lithium substances such as LiF, Li_3_N, and Li_2_O. These species are products of LiTFSI decomposition. Their presence plays a key role in enhancing ionic conductivity and forming an electron tunneling barrier at the interface between the CPSEs and the lithium anode. These effects reduce interfacial side reactions, promote uniform lithium plating and stripping, and ultimately form a dense and stable SEI layer that inhibits the growth of lithium dendrites. However, we note that the contribution of OGDY to the generation of inorganic lithium is limited. The contents of inorganic lithium species LiF, Li_3_N, and Li_2_O are comparable in the SEI layer on the lithium anode surface of Li|PEO|Li and Li|OGDY/PEO|Li.





**Fig. S16** Linear scanning voltammetry (LSV) curves of PEO and OGDY/PEO membranes

The linear scanning voltammetry (LSV) tests revealed that the electrochemical stabilization window of the OGDY/PEO CPSEs reached 5.0 V, which is a significant improvement compared to the electrochemical stabilization window of 4.8 V of the pure PEO PSEs. This result indicates that OGDY/PEO has a wider range of electrochemical stability and can be matched with a wider variety of cathode materials, thus providing greater flexibility in battery design.





**Fig. S17** Charge/discharge voltage profiles Li|PEO|LFP at 0.5 C


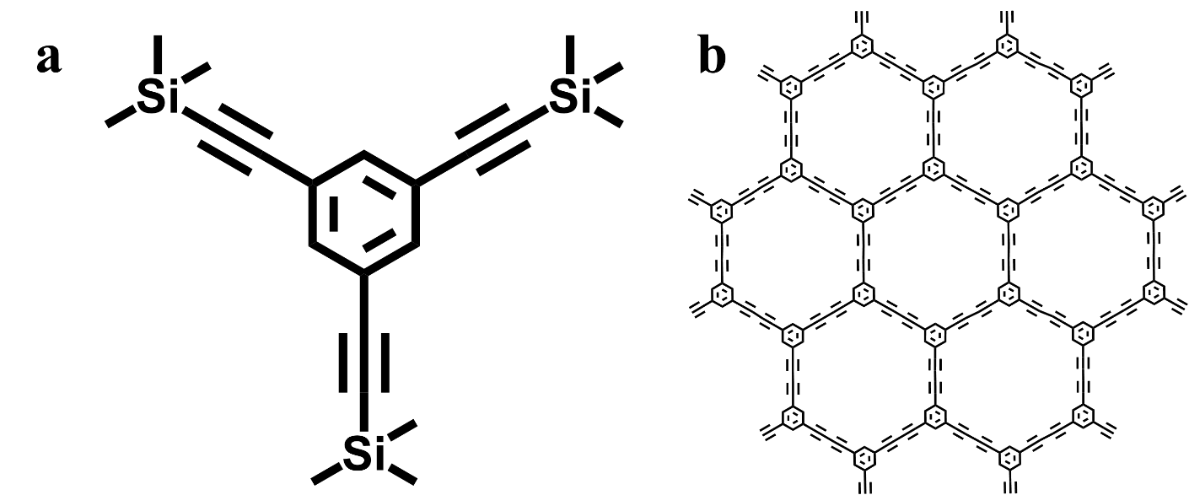


**Fig. S18** (**a**) The structure of HGDY monomer. (**b**) The structure of HGDY


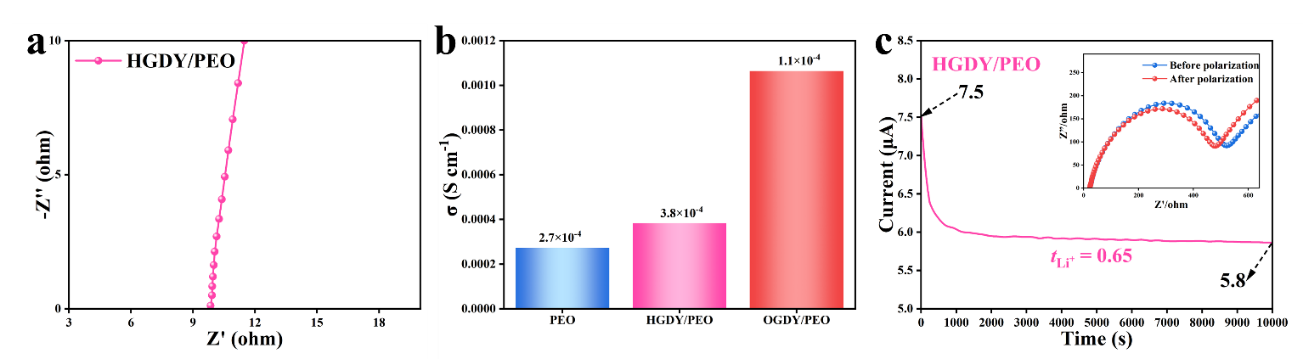


**Fig. S19** (**a**) EIS of HGDY/PEO blocked cells at 60℃. (**b**) σ of PEO, HGDY/PEO, and OGDY/PEO. (**c**) Chronoamperometry curve of Li|HGDY/PEO|Li symmetric cell under 10 mV polarization. Inset: the EIS before and after potential polarization


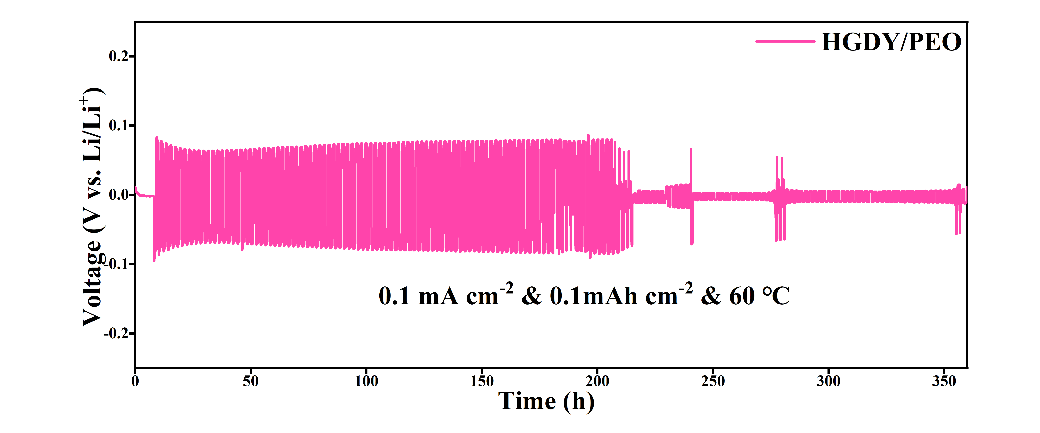


**Fig. S20** Long-term cycle performance of Li|HGDY/PEO|Li symmetric cells at 60 ^o^C, 0.1 mA/cm^2^, and 0.1mAh/cm^2^


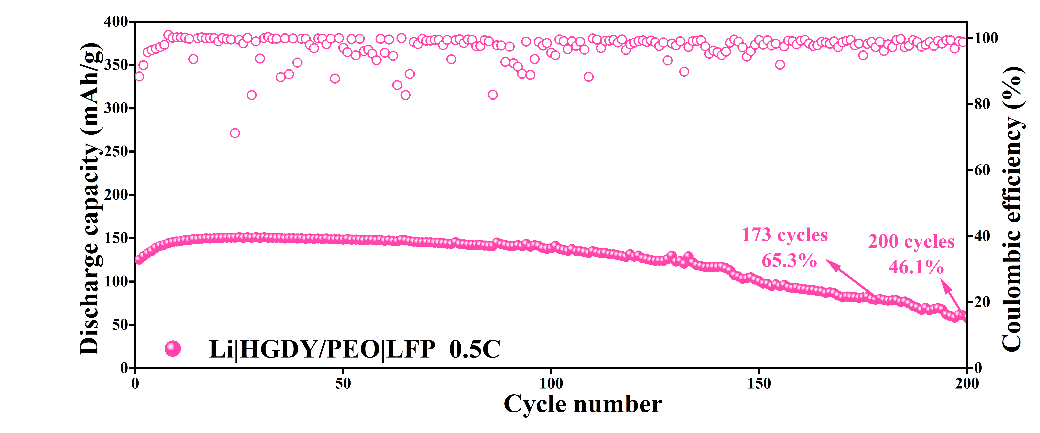


**Fig. S21** Charge/discharge voltage profiles Li|HGDY/PEO|LFP at 0.5 C

**Table S1** Parameters used by Slow-growth approach for OGDY and PEO

| Slow Growth Approach in  Migration Path | CV (Å) | Transformation velocity (Å∙fs^-1^) |
| --- | --- | --- |
| Path-PEO(from 3O-site to 3O-site) | d(Li_129_-O_40_)- d(Li_129_-O_36_)- d(Li_129_-O_37_) | -0.00172 (t=5000 fs) |
| 0O-path-OGDY(from Site1 to Site2) | d(Li_153_-C_108_) | -0.0017 (t=5000 fs) |
| 1O-path-OGDY(from Site1 to Site3) | d(Li_153_-O_45_) | -0.00151 (t=1000 fs) |
| 1O-path-OGDY(from Site3 to Site4) | d(Li_153_-C_62_) | -0.00135 (t=4000 fs) |
| 2O-path-OGDY(from Site1 to Site3) | d(Li_153_-O_45_) | -0.00151 (t=1000 fs) |
| 2O-path-OGDY(from Site3 to Site5) | d(Li_153_-O_48_) | -0.0002182 (t=4000 fs) |
| 2O-path-OGDY(from Site5 to Site6) | d(Li_153_-C_131_) | -0.0002018 (t=10000 fs) |

[a] The atomic labels in OGDY are shown in the figure below.


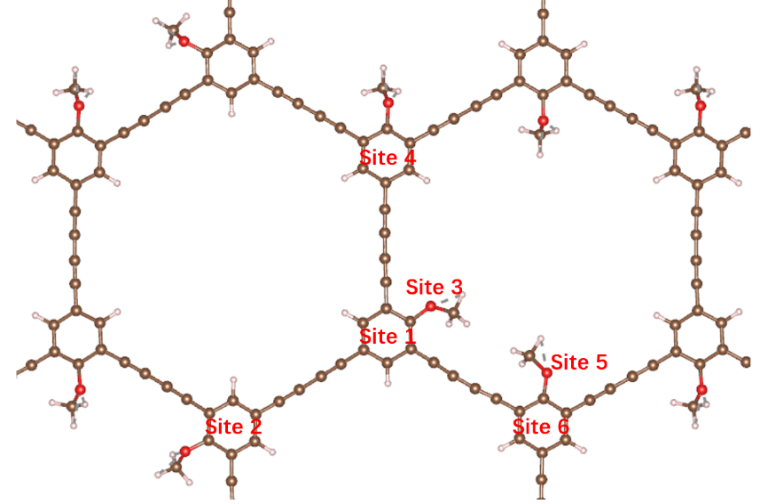


**Table S2** NPA charge of each atom in OGDY^[a]^

| No. | NPA charge | No. | NPA charge | No. | NPA charge |
| --- | --- | --- | --- | --- | --- |
| 1 | -0.08951 | 45 | -0.05077 | 89 | -0.09143 |
| 2 | -0.20309 | 46 | 0.07018 | 90 | 0.22353 |
| 3 | 0.42305 | 47 | 0.05648 | 91 | -0.15819 |
| 4 | -0.16667 | 48 | -0.06343 | 92 | -0.08964 |
| 5 | -0.0915 | 49 | -0.0683 | 93 | -0.09184 |
| 6 | -0.15622 | 50 | 0.07071 | 94 | -0.20352 |
| 7 | 0.22099 | 51 | 0.07211 | 95 | 0.21941 |
| 8 | 0.07643 | 52 | -0.06948 | 96 | -0.16578 |
| 9 | -0.05512 | 53 | -0.08926 | 97 | 0.22379 |
| 10 | -0.05235 | 54 | -0.14308 | 98 | 0.42379 |
| 11 | 0.06607 | 55 | 0.22623 | 99 | 0.22672 |
| 12 | 0.07023 | 56 | -0.16513 | 100 | 0.22667 |
| 13 | -0.06774 | 57 | -0.09261 | 101 | -0.51733 |
| 14 | -0.09035 | 58 | 0.42264 | 102 | -0.55255 |
| 15 | -0.1408 | 59 | -0.15617 | 103 | -0.19022 |
| 16 | 0.06185 | 60 | 0.22344 | 104 | 0.18578 |
| 17 | -0.07043 | 61 | -0.20262 | 105 | 0.18046 |
| 18 | -0.06958 | 62 | -0.09103 | 106 | 0.17494 |
| 19 | 0.0595 | 63 | 0.22069 | 107 | -0.18584 |
| 20 | -0.16916 | 64 | 0.07136 | 108 | 0.18366 |
| 21 | 0.41019 | 65 | -0.06908 | 109 | 0.16817 |
| 22 | -0.09935 | 66 | -0.08979 | 110 | 0.16845 |
| 23 | -0.1688 | 67 | 0.0631 | 111 | 0.22369 |
| 24 | -0.143 | 68 | -0.07111 | 112 | 0.22257 |
| 25 | -0.09938 | 69 | -0.06907 | 113 | -0.51449 |
| 26 | 0.22261 | 70 | 0.05933 | 114 | -0.51572 |
| 27 | 0.06498 | 71 | -0.14221 | 115 | -0.19056 |
| 28 | -0.05308 | 72 | 0.22644 | 116 | 0.1751 |
| 29 | -0.06432 | 73 | -0.51863 | 117 | 0.181 |
| 30 | 0.07092 | 74 | -0.52224 | 118 | 0.18742 |
| 31 | -0.06482 | 75 | -0.1895 | 119 | -0.19079 |
| 32 | -0.09024 | 76 | 0.18371 | 120 | 0.18105 |
| 33 | -0.13896 | 77 | 0.17984 | 121 | 0.17529 |
| 34 | 0.06963 | 78 | 0.17469 | 122 | 0.1879 |
| 35 | -0.09072 | 79 | -0.18995 | 123 | 0.07737 |
| 36 | -0.15559 | 80 | 0.18013 | 124 | -0.05906 |
| 37 | -0.09369 | 81 | 0.18534 | 125 | -0.08917 |
| 38 | -0.16705 | 82 | 0.17457 | 126 | -0.14018 |
| 39 | 0.4217 | 83 | -0.20301 | 127 | 0.22654 |
| 40 | -0.19874 | 84 | -0.08906 | 128 | 0.07833 |
| 41 | 0.22215 | 85 | 0.42379 | 129 | -0.06023 |
| 42 | 0.22295 | 86 | -0.15751 | 130 | -0.08871 |
| 43 | 0.07474 | 87 | 0.22046 | 131 | -0.14137 |
| 44 | -0.05459 | 88 | -0.16667 | 132 | 0.22636 |

[a] The atomic labels in OGDY are shown in the figure below.


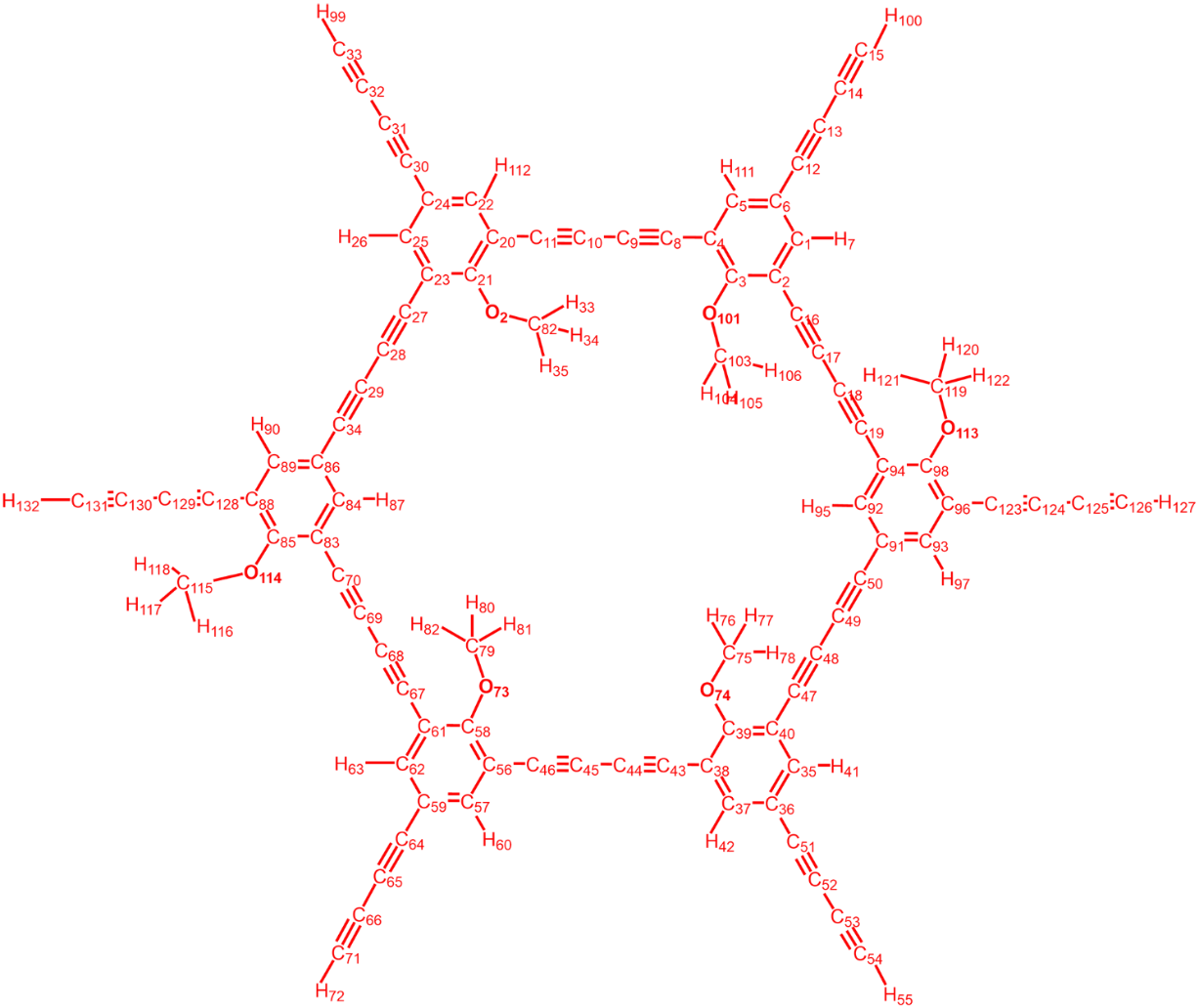


**Table S3** The σ of PEO and OGDY/PEO at different temperatures^[a]^

| Solid electrolyte | 30℃ | 40℃ | 50℃ | 60℃ | 70℃ |
| --- | --- | --- | --- | --- | --- |
| PEO | 4.2×10^-6^ | 1.3×10^-5^ | 4.7×10^-5^ | 2.7×10^-4^ | 3.9×10^-4^ |
| OGDY/PEO | 2.2×10^-5^ | 7.5×10^-5^ | 3.2×10^-4^ | 1.1×10^-3^ | 1.2×10^-3^ |

[a] The unit of σ is S cm^-1^.

**Table S4** Comparison of performance with literature batteries

| Solid electrolyte | Test condition | Initial specific capacity (mAh g^−1^) | Capacity retention rate | Cycles | σ (S cm^-1^) | t_Li+_ | References |
| --- | --- | --- | --- | --- | --- | --- | --- |
| MEMO@LLZTO-PEO-NF | 0.5 C, 60℃ | 104.3 | 63.7% | 200 | —— | 0.53 | [S2] |
| LFP/PEO-5 wt% 3DBN/Li | 1C, 60℃ | 138.9 | 89% | 100 | 8.9×10^-4^ | 0.21 | [S3] |
| LCPE-60 | 0.5C, 60℃ | 133 | 92.6% | 240 | 6.74×10^-4^ | —— | [S4] |
| PEO: LLZTO | 0.1C, 60℃ | 107 | 90% | 200 | 5.6×10^-4^ | 0.46 | [S5] |
| PAL | 0.2C, 60℃ | 142 | 91% | 200 | —— | 0.51 | [S6] |
| PPL | 1C, 60℃ | 146 | 66% | 500 | 1.54×10^-4^ | —— | [S7] |
| OV-LLZTO/PEO | 5C, 60℃ | —— | 75.68% | 500 | 5.6×10^-4^ | 0.328 | [S8] |
| CNF-COF@PEO | 0.2C, 60℃ | 137.2 | 97.2 | 500 | 6.34×10^−4^ | 0.81 | [S9] |
| PEO-POF | 1C, 60℃ | 133.6 | 96% | 300 | 5.32×10^−5^ | 0.64 | [S10] |
| PEO-Br-TPOM | 0.2C, 60℃ | 130 | 88% | 550 | —— | 0.53 | [S11] |
| OGDY/PEO | 0.5C, 60℃ | 158.7 | 91.4% | 205 | 1.1×10^-3^ | 0.71 | Our work |

**Table S5** Theσand interfacial impedance of OGDY/PEO at 80, 100, and 150 ℃

| Temperature | 80℃ | 100℃ | 150℃ |
| --- | --- | --- | --- |
| σ (S cm^-1^) | 1.6×10^-3^ | 2.2×10^-3^ | 4.2×10^-3^ |
| Surface resistance (ohm) | 5.13 | 3.68 | 1.98 |

Supplementary References

1. A. Du, H. Lu, S. Liu, S. Chen, Z. Chen et al., Breaking the trade-off between ionic conductivity and mechanical strength in solid polymer electrolytes for high-performance solid lithium batteries. Adv. Energy Mater. 14(31), 2400808 (2024). <https://doi.org/10.1002/aenm.202400808>
2. T. Duan, H. Cheng, Y. Liu, Q. Sun, W. Nie et al., A multifunctional Janus layer for LLZTO/PEO composite electrolyte with enhanced interfacial stability in solid-state lithium metal batteries. Energy Storage Mater. 65, 103091 (2024). <https://doi.org/10.1016/j.ensm.2023.103091>
3. Y. Ma, J. Wu, H. Xie, R. Zhang, Y. Zhang et al., The synthesis of three-dimensional hexagonal boron nitride as the reinforcing phase of polymer-based electrolyte for all-solid-state Li metal batteries. Angew. Chem. Int. Ed. 63(13), e202317256 (2024). <https://doi.org/10.1002/anie.202317256>
4. X. Zhang, C. Fu, S. Cheng, C. Zhang, L. Zhang et al., Novel PEO-based composite electrolyte for low-temperature all-solid-state lithium metal batteries enabled by interfacial cation-assistance. Energy Storage Mater. 56, 121–131 (2023). <https://doi.org/10.1016/j.ensm.2022.12.048>
5. J. Zhang, N. Zhao, M. Zhang, Y. Li, P.K. Chu et al., Flexible and ion-conducting membrane electrolytes for solid-state lithium batteries: Dispersion of garnet nanoparticles in insulating polyethylene oxide. Nano Energy 28, 447–454 (2016). <https://doi.org/10.1016/j.nanoen.2016.09.002>
6. X. Da, J. Chen, Y. Qin, J. Zhao, X. Jia et al., CO_2_-assisted induced self-assembled aramid nanofiber aerogel composite solid polymer electrolyte for all-solid-state lithium-metal batteries. Adv. Energy Mater. 14(11), 2303527 (2024). <https://doi.org/10.1002/aenm.202303527>
7. J. Wu, Z. Rao, Z. Cheng, L. Yuan, Z. Li et al., Ultrathin, flexible polymer electrolyte for cost-effective fabrication of all-solid-state lithium metal batteries. Adv. Energy Mater. 9(46), 1902767 (2019). <https://doi.org/10.1002/aenm.201902767>
8. Y. Fu, K. Yang, S. Xue, W. Li, S. Chen et al., Surface defects reinforced polymer-ceramic interfacial anchoring for high-rate flexible solid-state batteries. Adv. Funct. Mater. 33(10), 2210845 (2023). <https://doi.org/10.1002/adfm.202210845>
9. X. Yang, L. Fang, J. Li, C. Liu, L. Zhong et al., Multipolar conjugated polymer framework derived ionic sieves *via* electronic modulation for long-life all-solid-state Li batteries. Angew. Chem. Int. Ed. 63(23), e202401957 (2024). <https://doi.org/10.1002/anie.202401957>
10. W. Liang, X. Zhou, B. Zhang, Z. Zhao, X. Song et al., The versatile establishment of charge storage in polymer solid electrolyte with enhanced charge transfer for LiF-rich SEI generation in lithium metal batteries. Angew. Chem. Int. Ed. 63(18), e202320149 (2024). <https://doi.org/10.1002/anie.202320149>
11. X. Zhou, F. Huang, X. Zhang, B. Zhang, Y. Cui et al., Interface-targeting carrier-catalytic integrated design contributing to lithium dihalide-rich SEI toward high interface stability for long-life solid-state lithium-metal batteries. Angew. Chem. Int. Ed. 63(21), e202401576 (2024). <https://doi.org/10.1002/anie.202401576>
